# An overview of CAR T-cell clinical trial activity to 2021

**DOI:** 10.1093/immadv/ltab004

**Published:** 2021-03-09

**Authors:** Antonella Adami, John Maher

**Affiliations:** 1 King’s College London, School of Cancer and Pharmaceutical Sciences, CAR Mechanics Lab, Guy’s Cancer Centre, London, UK; 2 Department of Clinical Immunology and Allergy, King’s College Hospital NHS Foundation Trust, London, UK; 3 Department of Immunology, Eastbourne Hospital, Kings Drive, East Sussex, UK; 4 Leucid Bio Ltd., Guy’s Hospital, London, UK

**Keywords:** cancer, immunotherapy, CAR T-cell, clinical trials

## Abstract

Immunotherapy of cancer using chimeric antigen receptor-engineered T-cells has transformed the management of selected haematological malignancies, triggering intense clinical trial activity in this arena. This article summarises trial activity that has been published to date across the spectrum of haematological and solid tumour types.

In recent years, we have witnessed a paradigm shift in cancer treatment with the advent of effective immunotherapies for both haematological and solid cancers. Alongside traditional therapeutic modalities, immune checkpoint inhibitors, and cell-based therapies are increasingly being used in mainstream clinical practice. In what would have been an unthinkable development some 20 years ago, cell-based immunotherapies have now become the largest area of drug development in immuno-oncology. Moreover, it is particularly noteworthy that the largest year on year increase in this activity has involved the Chimeric antigen receptors (CAR) T-cell sector [1].

CAR T-cells are synthetic receptors that bind one or more native cell surface target(s), obviating the need for human leukocyte antigen (HLA)-dependent antigen presentation or restriction. While this limits targeting potential to the subset of proteins found on the cell surface, CAR specificity can also be directed against non-proteinaceous antigens (e.g. tumour-associated gangliosides) or peptide/HLA complexes. The first CAR was described over 30 years ago and entailed the substitution of the variable domains of an antibody heavy and light chain for the corresponding regions within an α β T-cell receptor (TCR) heterodimer [2]. Eshhar simplified this design to create a homodimeric fusion receptor, cleverly using a single-chain antibody fragment to confer target specificity [3]. This antigen recognition element was fused to a spacer, transmembrane domain, and TCR-like activating domain, thereby coupling tumour engagement to the delivery of a cytotoxic T-cell signal. However, these early ‘first-generation’ designs failed to achieve clinical impact and CAR T-cell research remained a niche and somewhat dismissed academic activity for many years. This situation was dramatically reversed through the introduction of a co-stimulatory domain within the linear CAR framework, a platform that was conceived and first evaluated by Finney and colleagues in the Jurkat leukaemic T-cell model [4]. Two clinically active second-generation CAR systems have been developed in which either CD28 or 4-1BB are used to provide co-stimulation, while the ζ chain of the TCR-associated CD3 complex is most commonly employed to deliver an activating signal.

Clinical development of CAR T-cell immunotherapy was pioneered in the USA, at a speed reminiscent of the space race. Intense clinical trial activity at multiple centres was initiated on the back of encouraging early case reports which demonstrated the therapeutic promise of CD19-specific second-generation CAR T-cells against B-cell malignancy [5, 6]. The fruit of these collective efforts was the attainment of unprecedented response rates in patients with relapsed/ refractory B-cell leukaemias and a range of lymphomas. Commercialisation soon followed as major academic centres partnered with the pharmaceutical industry, or established successful new companies. Ultimately, this led to marketing approval in the USA of three CD19-specific CAR T-cell products for the treatment of relapsed/ refractory B-cell malignancy, namely Tisagenlecleucel (Kymriah), Axicabtagene Ciloleucel (Yescarta), and Brexucabtagene autoleucel (Tecartus). Both Kymriah and Yescarta have also secured regulatory approval in many other territories worldwide, including the UK. Further approvals are highly likely in the near future.

Given the ever-increasing number of clinical trials involving CAR T-cell immunotherapy of cancer, we set out here to capture published studies in a simple tabular format. Had this task been undertaken 10 years ago, a very short list indeed would have been generated. In total, we identified more than 150 CAR T-cell clinical trials that involved a minimum of six patients (published in abstract or full manuscript format) and these are presented chronologically in Supplementary Table 1. Our overview is designed to complement a presentation of the therapeutic landscape in this area, published last year [7]. Wherever possible, data has been selected from peer previewed publications rather than entries made on other sites. We also sought to extract information from the newest articles pertaining to each trial and have employed commonly used abbreviations for diseases, as detailed at the foot of the Table. Pivotal (registrational) trials are highlighted as grey rows to distinguish these form earlier phase studies. While the table is dominated by trials involving B-cell malignancy, there are an increasing number of studies focussed on multiple myeloma, in which B-cell maturation antigen has proved to be a highly tractable target. We have presented response and survival data in addition to the two common acute toxicities that are associated with this intervention, namely cytokine release syndrome and immune effector cell‐associated neurotoxicity. In reviewing the list, readers may wish to review the original trial data since there is considerable variability across studies in the use of ancillary or bridging therapies. Patient conditioning with lymphodepleting chemotherapy was undertaken in the majority of trials and there is a clear preference for the use of fludarabine and cyclophosphamide in this role [8].

Solid tumours represent the bulk of human cancers and represented the primary focus of early CAR T-cell clinical trial activity. However, even with the advent of second-generation CAR systems, we have only seen sporadic responses in a small number of patients. While the list of targets under study in B-cell and plasma cell malignancy is limited, there is much greater diversity in target selection in solid tumour-based clinical trials. This sub-optimal efficacy justifies the need for additional innovation around CAR design, the nature and fitness of the cellular host, and complementary use of additional interventions that may help to render the tumour microenvironment more favourable.

An important enabling attribute of CAR T-cell technology is the tremendous potential for innovation around this highly modular framework. Illustrating this, we are seeing the emergence of many switchable systems in which a universal CAR-engineered cell is utilised in combination with a bridging molecule that confers the desired tumour antigen specificity. A range of drug controllable, logic-gated, and split-signalling CAR systems are also in development that offer the potential to improve therapeutic specificity and control. A further key point is the compatibility of CAR technology with other cutting-edge innovations. These include genome editing technologies, systems to precisely modulate host cell gene expression, cytokine armouring approaches and potential for metabolic manipulation and modulation of epigenetic properties of host cells.

The landscape of clinical CAR T-cell immunotherapy continues to evolve rapidly. Recently, there has been a tremendous expansion of clinical CAR T-cell activity in China, which overtook the USA in 2017 as the country with the greatest number of registered CAR T-cell clinical trials [9]. A cursory inspection of the accompanying table emphasises this evolving trend. Alternative cell hosts other than α β T-cells are increasingly being studied and are now beginning to emerge as viable alternatives for clinical use. Examples include natural killer (NK) cells, γδ T-cells, invariant NK T-cells, and macrophages. In an increasing number of cases, these cells are being used as the basis to develop an allogeneic or off-the-shelf CAR therapy. A further exciting example involves the use of induced pluripotent stem cells, a technology that offers the promise of unprecedented scalable manufacture, although safety will need to be strictly ensured.

Immunotherapy using CAR-engineered immune cells has emerged as a transformative therapeutic intervention for human cancer and is now increasingly under study in other disease arenas, such as autoimmunity and transplantation. We look forward to the increasing clinical translation of these flexible and transformative therapies in the coming years.

## Supplementary Material

ltab004_suppl_Supplementary_Table_1

## Data Availability

Data derived from public domain sources.

## Abbreviations

CAR: Chimeric antigen receptors
NK: Natural killer
TCR: T-cell receptor
