# Supplementary material for: An overview of CAR T-cell clinical trial activity to 2021
Source: Immunother Adv. 2021 Mar 9;1(1):ltab004. doi: 10.1093/immadv/ltab004 (PMC8137996; doi:10.1093/immadv/ltab004)
Supplement: ltab004_suppl_Supplementary_Table_1 [file ltab004_suppl_supplementary_table_1.docx]

| **Ref** | **Year** | **Institution/**  **Sponsor** | **Clinical Trial register** | **Target** | **CAR Gen.** | **Disease** | **No.**  **treated** | **Conditioning** | **Cell product & dose** | **Clinical**  **outcome** | **Toxicity (>grade 3)** | **Survival** |
| --- | --- | --- | --- | --- | --- | --- | --- | --- | --- | --- | --- | --- |
| ***B-cell malignancy*** | | | | | | | | | | | | |
| (1) | 2011 | MSKCC | NCT00466531  NCT01044069 | CD19 | 2G: CD28 | B-CLL  B-ALL | 9 | Nil or Cy | 1.2-3x10^7^/Kg (B-CLL)  3x10^6^, 1-3x10^7^/Kg (B-ALL) | 33% SD | 0% CRS  0% ICANS  1 fatality - presumed sepsis | N/A |
| (2) | 2011 | Baylor | N/A | CD19 | 2G: CD28 | B-NHL | 6 | Nil | 2x10^7^/m^2^  1x10^8^/m^2^  2x10^8^/m^2^ | 33% SD | 0% CRS  0% ICANS | SD n=2 for 3 and 10m |
| (3) | 2012 | NCI | NCT00924326 | CD19 | 2G: CD28 | B-NHL  B-CLL | 8 | Flu/Cy | 0.3-3x10^7^/Kg | 13% CR  75% PR | >Grade 3 toxicity in all patients | 1 ongoing CR at 15m |
| (4) | 2014 | MSKCC | NCT01044069 | CD19 | 2G: CD28 | B-ALL | 16 | Cy | 3x10^6^/Kg | 88% CR/CRi | 44% CRS  6% ICANS | 44% in remission post allo HSCT |
| (5) | 2014 | Novartis | NCT01626495  NCT01029366 | CD19 | 2G: 41BB | B-ALL  Children/  young adults | 30 | Flu/Cy | 0.76x10^6^ to 20.6x10^6^/Kg | 90% CR | 27% CRS  0% ICANS | At 6m, EFS 67% |
| (6) | 2015 | UPenn | NCT01029366 | CD19 | 2G: 41BB | B-CLL | 14 | Flu/Cy/others | 0.14x10^8^ to 11x10^8^/Kg | 57% ORR  29% CR  29% PR | 35% CRS  7% ICANS | 100% PFS for CR pts - median duration 40m |
| (7) | 2015 | NCI | NCT00924326  (update of (3)) | CD19 | 2G: CD28 | B-NHL  B-CLL | 15 | Flu/Cy | 1x10^6^/Kg | 53% CR  27% PR | Seen in 13, including sepsis, CRS and ICANS | At 7m,  all CRs ongoing |
| (8) | 2015 | NCI | NCT01593696 | CD19 | 2G: CD28 | B-ALL  Children/  young adults | 20 | Flu/Cy | 1-3 x10^6^/Kg | 70% ORR  60% CR | 30% CRS  5% ICANS | 51.6% OS  med 10m follow-up |
| (9) | 2016 | Baylor | NCT00881920 | κ light chain | 2G: CD28 | B-NHL  B-CLL | 9 | Cy unless lymphopenic | 1.7x10^7^ to 1.9x10^8^/m^2^ | 2 CR and 1 PR | 0% CRS  0% ICANS | 1 durable CR at 3y |
| (10) | 2016 | Chinese PLA General Hospital | NCT01735604 | CD20 | 2G: 41BB | B-NHL | 11 | Cytoreductive chemotherapy, including Cy | 0.41x10^7^ to 1.46x10^7^/Kg | 82% ORR  55% CR | 0% CRS  0% ICANS | Med PFS >6m |
| (11) | 2016 | UPenn | NCT01747486 | CD19 | 2G: 41BB | B-CLL | 41 | Flu/Cy | 5x10^7^  5x10^8^ cells | 24% CR at higher dose | 20% CRS | Med follow-up 26m - 12% CR |
| (12) | 2016 | UPenn | NCT01626495 | CD19 | 2G: 41BB | B-ALL  (children) | 59 | Flu/Cy | 1x10^7^ to 1x10^8^/Kg | 93% CR | 27% CRS | Med follow-up 12m - 57% CR  79% OS |
| (13) | 2016 | City of Hope | NCT01318317 | CD19 | 1G | B-NHL | 8 | Administered post auto HSCT | 25 to 200x10^6^ (CD8^+^ CM) | 38% CR 25% PR | 0% CRS  0% ICANS | 50% PFS at 1y |
| (13) | 2016 | City of Hope | NCT01815749 | CD19 | 2G: CD28 | B-NHL | 8 | Administered post auto HSCT | 50 to 800x10^6^ (CD4^+^ and CD8^+^ CM) | 75% CR  25% PR | 0% CRS  0% ICANS | 75% PFS  at 1y |
| (14) | 2016 | FHCRC | NCT01865617 | CD19 | 2G: 41BB | B-ALL | 30 | Cy ± Flu | 2x10^5^/Kg  2x10^6^/Kg  2x10^7^/Kg  1:1 CD4:CD8 | 23% CRS  50% ICANS | 93% ORR  86% CR | 10% CR 400d post CAR-T |
| (15) | 2016 | FHCRC | NCT01865617 | CD19 | 2G: 41BB | B-NHL | 32 | Cy ± Flu | 2x10^5^/Kg  2x10^6^/Kg  2x10^7^/Kg  1:1 CD4:CD8 | 13% CRS  28% ICANS | Cy/Flu: 72% ORR, 50% CR  Cy: 50% ORR  8% CR | Med OS - No Flu 6.3m; Cy/Flu 25m  Med PFS -  No Flu 1.5m; Cy/Flu 5.8m |
| (16) | 2016 | MDACC | NCT00968760 | CD19 | 2G: CD28 | B-ALL  B-NHL | 7 | Auto HSCT | 10^7^ to 5x10^9^/m^2^  Sleeping beauty | 0% CRS  0% ICANS | 86% CR | 83.3% PFS at 30m |
| (17) | 2017 | UPenn | NCT02640209 | CD19 | 2G: 41BB | B-CLL | 10 | Flu/Cy | N/A | 89% CR (marrow) | 10% CRS | Med follow-up 6m |
| (18) | 2017 | UPenn | NCT02030834 | CD19 | 2G: 41BB | DLBCL  FL | 28 | Flu/Cy | 5x10^8^/Kg | 64% ORR  43% CR (DLBCL)  71% CR (FL) | 18% CRS  11% ICANS | Med  follow-up  28.6m  86% (DLBCL)-89% (FL) sustained  remission |
| (19) | 2017 | FHCRC | NCT02028455 | CD19 | 2G: 41BB | B-ALL  (paediatric/  young adults | 43 | Various, including Flu/Cy | 0.5-10x10^6^  0.5-5x10^6^/Kg | CR 93% | 93% CRS  49% ICANS | 51% EFS and  69% OS at 12m |
| (20) | 2017 | FHCRC | NCT01865617 | CD19 | 2G: 41BB | B-CLL | 24 treated  19 evaluable for efficacy | Flu/Cy | 2x10^5^/Kg  2x10^6^/Kg  2x10^7^/Kg  1:1 CD4:CD8 | 71% ORR | 83% CRS  33% ICANS | Med OS  6.6m |
| (21) | 2017 | Zhejiang University/ Innovative Cellular Therapeutics | ChiCTR-OCC-15007008 | CD19 | 2G: 41BB | B-ALL | 15 | Flu/Cy | 1.1 x10^6^ to 9.8x10^6^/Kg | 80% CR | 40% CRS  33% ICANS | OS 65.5% and  DFS 37.8% at 150d |
| (22) | 2017 | NCI | NCT00924326 | CD19 | 2G: CD28 | DLBCL | 22 | Flu/Cy | 1x10^6^ to  6x10^6^/Kg | 73% ORR  55% CR  18% PR | 0% CRS  55% ICANS | Duration of response 7-24m  CRs ongoing |
| (23) | 2017 | Hebei Yanda Lu Daopei Hospital / Shanghai Pulmonary Hospital | ChiCTR-IIh-16008711 | CD19 | 2G: 41BB | B-ALL | 51 | Flu/Cy | 0.05-14x10^5^ /Kg  1x10^5^/Kg | 90% CR/CRi | 16% CRS  4% ICANS | 23/27 bridged to allo HSCT remained in CR at med follow up of 206d |
| (24) | 2018 | MSKCC | NCT0144069 | CD19 | 2G: CD28 | B-ALL | 53 | Flu/Cy | 1x10^6^ to  3x10^6^/Kg | 83% CR | 26% CRS  43% ICANS | Med EFS 6.1m Med OS 12.9m |
| (25) | 2018 | NCI/ Stanford | NCT02315612 | CD22 | 2G: 41BB | B-ALL  children/  young adults | 21  (17 prior CD19 CAR-T) | Flu/Cy | 3x10^5^  to 3x10^6^/Kg | 73% CR | 0% CRS  28% ICANS (?grade) | Med response duration 6m |
| (26) | 2018 | Uppsala University | NCT02132624 | CD19 | 3G: CD28/  41BB | CD19^+^ leukaemia or lymphoma | 15 | Flu/Cy | 2x10^7^  to 2x10^8^  cells/m^2^ | 36% CR (B-NHL)  50% CR (B-ALL) | 20% CRS  13% ICANS | Med response duration 5m |
| (27) | 2018 | Sheba Medical Center | NCT02772198 | CD19 | 2G: CD28 | B-ALL | 20 | Flu/Cy | 1x10^6^/Kg | 90% CR | 20% CRS (grade 2-3)  30% ICANS (grade 2-4) | 73% EFS and  90% OS at 1y |
| (28) | 2018 | Third Military Medical University | NCT02349698 | CD19 | 2G: CD28  2G: 41BB | B-ALL | 10  (5-CD28)  (5-41BB) | Flu/Cy | 1x10^6^  to 1x10^7^/Kg | CD28: 60% CR  41BB: 60% CR | 0% CRS  0% ICANS | Med PFS 6 m |
| (29) | 2018 | Xuzhou Medical University | N/A | CD19 | 2G: 41BB | B-ALL | 14 | Flu/Cy | 1x10^6^/Kg | 92.9% CR/CRi | 29% CRS  7% ICANS (?grade) | At 180d  OS 65.8%  DFS 71.4% |
| (30, 31) | 2018 | Novartis | NCT02435849  (ELIANA) | CD19 | 2G: 41BB  (Kymriah; Tisagen-lecleucel) | B-ALL  (paediatric/  young adults | 79 | Flu/Cy in 76/79 | 0·2–5 × 10⁶  0·1–2·5x10⁸/Kg | 60% CR  21% CRi | 48% CRS  13% ICANS | 66% DFS at 18m  Med OS not reached |
| (32) | 2018 | Third Military Medical University | NCT02685670 | CD19 | 2G: CD28  2G: 41BB | B-ALL (6) and B-CLL (1) | 7 | Flu/Cy | 1x10^6^/Kg  2G CAR T-cells mixed 1:1 | 71% CR | 0% CRS  0% ICANS | Med OS 12m  Med PFS 5m |
| (33) | 2018 | Novartis-UPenn | NCT02445248  (JULIET) | CD19 | 2G: 41BB  (Kymriah; Tisagen-lecleucel) | DLBCL | 93 | Flu/Cy | Med dose 3x10^8^ | 52% ORR  40% CR  12% PR | 22% CRS  12% ICANS | DFS 65% at 12m |
| (34) | 2018 | MSKCC | NCT01416974 | CD19 | 2G: CD28 | B-CLL | 8 | Cy | 3x10^6^ to  3x10^7^/Kg | 25% CR  13% PR | 0% CRS  0% ICANS | Med PFS 13.6m  Med OS not reached |
| (35) | 2018 | Stanford University | NCT03233854 | CD19 & CD22 | 2G: 41BB  Bispecific CAR | B-ALL  DLBCL | 6 | Flu/Cy | 1x10^6^/Kg  3x10^6^/Kg  1x10^7^/Kg | 33% CR | 33% CRS  0% ICANS | N/A |
| (36) | 2018 | Eureka Therapeutics | NCT02658929  (ARTEMIS) | CD19 | 2G: 41BB | B-NHL | 21 | Flu/Cy | 1x10^6^/Kg  3x10^6^/Kg  6x10^6^/Kg | 52% ORR  29% CR | 0% CRS  0% ICANS | 24% CR at 6m |
| (37) | 2019 | Kite/ Gilead | NCT02348216  (ZUMA-1) | CD19 | 2G: CD28  (Yescarta; Axicabta-gene Ciloleucel) | B-NHL | 108 treated  101 evaluable for efficacy | Flu/Cy | 2x10^6^/Kg | 83% ORR  58% CR | 11% CRS  32% ICANS | Med duration of response 11.1m  Med PFS 5.9m |
| (38) | 2019 | MSKCC | NCT01840566 | CD19 | 2G: CD28 | B-NHL | 15 | Auto HSCT | 5x10^6^/Kg  1x10^7^/Kg | 53% CR | 40% CRS  67% ICANS | 30% PFS at  2y |
| (39) | 2019 | FHCRC | NCT01865617 | CD19 | 2G: 41BB | B-ALL | 57 treated  53 evaluable for efficacy | Flu/Cy | 2x10^6^/Kg  1:1 CD4:CD8 | 85% CR | N/A | Med follow-up  30.9m  EFS 61%  OS 72% |
| (40) | 2019 | FHCRC | NCT01865617 | CD19 | 2G: 41BB | B-NHL | 65 treated  47 evaluable  For efficacy | Flu/Cy | 2x10^6^/Kg  1:1 CD4:CD8 | 51% ORR  40% CR | N/A | Med PFS 20m in CR patients |
| (41) | 2019 | UCL | NCT02443831  (CARPALL) | CD19 | 2G: 41BB  AUTO1 | B-ALL  Burkitt NHL  (paediatric/  young adults) | 14 | Flu/Cy | 1x10⁶ cells/Kg | 86% CR | 0% CRS  7% ICANS | OS 84% at 6m  OS 63% at 12m |
| (42) | 2019 | Shanghai Institute of Hematology | NCT03355859 | CD19 | 2G: 41BB | B-NHL | 10 treated  9 evaluable for efficacy | Flu/Cy | 2x x10^7^, 5 x10^7^  and 1 x10^8^ cells | 67% CR  33% PR | 0% CRS  11% ICANS | 56% still in CR at 6m |
| (43) | 2019 | FHCRC | NCT01865617 | CD19 | 2G: 41BB | B-NHL:  FL (38%)/  tFL(62%) | 21 | Flu/Cy | 2x10^6^ cells/Kg  1:1 CD4:CD8 | 88% CR (FL)  46% CR (tFL) | 0% CRS  0% ICANS | All FL CR patients still in CR at 24m; Med PFS 10.2m in tFL group |
| (44) | 2019 | MSKCC | NCT01860937 | CD19 | 2G: CD28 | B-ALL  (paediatric/  young adults) | 25 treated  24 evaluable | High or low dose Cy | 1x10^6^/Kg  3x10^6^/Kg | CR/CRi 75% | 16% CRS  28% ICANS | Most consolidated by allo HSCT. 44% alive at time of publication |
| (45) | 2019 | The Second Hospital of Hebei Medical University | NCT02963038 | CD19 | 2G: 41BB | B-ALL | 10 | Flu/Cy | Average dose 0.71x10^6^/Kg | 80% CR | 40% CRS  30% ICANS  (all grades) | Med OS 10.3m  Med EFS 4m |
| (46) | 2019 | MGH | N/A | CD19 | 2G: 41BB | CNS Lymphoma | 8 | Flu/Cy | 0.6x10^8^  to 6x10^8^/Kg | 25% CR  25% PR | 0% CRS  0% ICANS | 38% ongoing response at >180d |
| (47) | 2019 | Peking Univ Cancer Hospital; Norris Comp Cancer Center | NCT02842138 | CD19 | 2G: 41BB | B-NHL | 25 | Flu/Cy | 3x10^6^  to 3.6x10^8^ | 28% CR (55% at highest dose level); 32% PR | 0% CRS  0% ICANS | Med response >181d for 6 patients show achieved CR at highest dose level |
| (48) | 2019 | Huazhong University of Science and Technology | NCT02965092  and  NCT03366350 | CD19 | 2G: 41BB | B-ALL | 58 | Flu/Cy | 0.89x10^6^  to 4.01x10^6^/Kg | 87.9% CR | 38% CRS  15% ICANS | Consolidation by allo HSCT in 21 patients.  Med OS 16.1m |
| (49) | 2019 | Tianjin Medical University | ChiCTR-ONN-16009862 and ChiCTR1800019288 | CD19 | 2G: 41BB | B-NHL | 11 | Flu/Cy and nivolumab | 8x10^6^ /Kg | 82% ORR  45% CR | 0% CRS  0% ICANS | Med PFS 6m  27% ongoing CR |
| (50) | 2019 | Peking Univ Cancer Hospital | NCT03528421 | CD19 | 2G: CD28  2G: 41BB | B-NHL | 9  (CD28 3; 4-1BB 6) | Flu/Cy | 0.75-5x10^5^/Kg | 78% CR | 1/3 grade 5 CRS (CD28)  1/3 grade 3 ICANS (CD28) | 67% response ongoing at 3m |
| (51) | 2019 | MSKCC | NCT004466531 | CD19 | 2G: CD28 | B-CLL  B-NHL | 20 treated  12 CLL patients evaluable | Nil or Cy | 0.4-3x10^7^/Kg | 25% CR | 10% CRS  10% ICANS | Med follow-up at 53m, all CR stable |
| (52) | 2019 | Multicentre China | ChiCTR-OIC-17013523 | CD22 | 2G: 41BB | B-ALL | 34 treated  30 evaluable | Flu/Cy | ≤1 × 10^6^/kg to  ≤4 × 10^6^ per kg | 80% CR/CRi | 3% CRS  0% ICANS | 1-year LFS  71.6% (11 bridged to allo HSCT) |
| (53) | 2019 | Multicentre China | N/A | CD19 | 4G: CD28 +CD27 | B-ALL | 25 | Flu/Cy | Med dose  7.133 × 10^5^/kg | 92% ORR  80% CR | 0% CRS  0% ICANS | Med OS 267 |
| (54) | 2019 | Autolus | NCT03289455  (Amelia) | CD19 & CD22 | 2 x 2G: OX40  (CD19)  4-1BB  (CD22)  (AUTO3) | B-ALL  (paediatric/  young adults) | 10 treated  7 evaluable | Flu/Cy | 1,3 or 5x10^6^ /Kg | 100% CR/CRi | 0% CRS  0% ICANS | Med follow-up 8m  57% CR/CRi |
| (55) | 2020 | NCI | NCT00924326  (update of (3) and (7)) | CD19 | 2G: CD28 | B-NHL  B-CLL | 43 | Flu/Cy | 1-30x10^6^ /Kg | 81% ORR  58% CR | N/A | Med follow up 42m  Med EFS 55m |
| (56) | 2020 | Medical College of Wisconsin | NCT03019055 | CD19 & CD20 | 2G: 41BB  Bispecific CAR | B-NHL 19  B-CLL 3 | 22 | Flu/Cy | 2.5x10^5^  to 2.5x10^6^/Kg | 82% ORR  64% CR | 5% CRS  14% ICANS | Med OS 20.3m |
| (57) | 2020 | NCI | NCT02659943 | CD19 | 2G: CD28 | B-NHL | 20 | Flu/Cy | 0.66 × 10^6^, 2 × 10^6^ or 6 × 10^6^ cells/Kg  (some received 2 doses) | 70% OR  55% CR | 10% CRS  5% ICANS | 40% ongoing CRs ranging from 17-35m |
| (58) | 2020 | Kite/ Gilead | NCT02601313 (ZUMA-2) | 2 | 2G: CD28  (Tecartus; Brexucab-tagene autoleucel) | Mantle cell  lymphoma | 68 treated  60 evaluable for efficacy | Flu/Cy | 2x10^6^ cells/Kg | 67% CR  27% PR | 15% CRS  31% ICANS | At 12m PFS 61% and OS 81% |
| (59) | 2020 | Lu Daopei Hospital | NCT03173417 | 1/2 | 2G: CD28  2G: 41BB | B-ALL  65% children  35% adult | CD28 21  4-1BB 89 | Flu/Cy | 1-10x10^5^/Kg | 93% CR | 16% CRS  14% ICANS (grade 2-3) | 58% DFS and  64% OS at 1y |
| (60) | 2020 | Autolus | NCT03287817  (Alexander) | CD19 & CD22 | 2 x 2G: OX40  (CD19)  4-1BB  (CD22)  (AUTO3) | DLBCL | 19 treated  18 evaluable for efficacy | Flu/Cy & Pembro | 50,150 or 450x10^6^ cells | dose > 50 x 10^6^  64% ORR  55% CR | 0% CRS  5% ICANS | All CRs ongoing at 1-12m |
| (61) | 2020 | Chinese PLA General Hospital | NCT03185494 | CD19 & CD22 | 2G: 41BB  Bispecific CAR | B-ALL | 6 | Flu/Cy | 1.7x10^6^  To 3x10^6^/Kg | 100% CR | 0% CRS  0% ICANS | DFS  3-11m |
| (62) | 2020 | UPenn | NCT02030847 and NCT01029366 | CD19 | 2G: 41BB | B-ALL  (adult) | 35 | Flu/Cy | 5×10^7^ cells (9)  5×10^8^ cells (26) (single dose or fractionated) | 33% CR low dose  90% CR high dose | 3 CRS deaths in high single dose group;  High dose fractionated safest and most effective with 5% CRS | High dose fractionated 2y OS 73%  EFS 49.5% |
| (63) | 2020 | Multicentre China | ChiCTR- OOC-16007779. | CD19 | 4G: CD28 +CD27 | B-NHL | 21 | Flu/Cy | 8.9x10^5^/Kg | 67% OR  43% CR  24% PR | 0% CRS  5% ICANS | Med OS 23.8m |
| (64) | 2020 | FHCRC | NCT01865617 | CD19 | 2G: 41BB  (outcome of second infusion) | B-ALL 14  B-CLL 9  B-NHL 21 | 44 | Flu/Cy | 2 doses  CART1&2 ALL  2x10^5^ to 2x10^6^/Kg  CART1&2 CLL/NHL  2x10^6^ to 2x10^7^/Kg | B-ALL 21% CR  B-CLL 22% CR  B-NHL 19% CR | 9% CRS  11% ICANS | Med duration response  B-CLL, B-NHL, B-ALL was 33,6 and 4m respectively |
| (65) | 2020 | JW Therapeutics  Shanghai Ming Ju Biotechnol. Company | NCT04089215 | CD19 | 2G: 41BB  Relmacab-tagene autoleucel | B-NHL | 59 treated  58 evaluable for efficacy | Flu/Cy | 100x10^6^  150x10^6^ | 76% ORR  52% CR | 5.1% CRS  5.1% ICANS | ORR 60.3% at 3m |
| (66) | 2020 | Multicentre China | ChiCTR-OIB-17013670 | CD19 & CD22 | 2G: 41BB  Sequential CAR T-cell infusions | B-ALL (paediatric) | 20 | Flu/Cy | Median dose for both CAR T-cells 10x10^5^/Kg | 100% CR | 5% CRS  5% ICANS | DFS and OS 79.5% and 92.3% respectively at 1y (no HSCT consolidation) |
| (67) | 2020 | Second Hospital of Anhui Medical University | NCT02735291 | CD19 | 2G: 41BB | B-ALL  (paediatric/  adult) | 51 treated  47 evaluable for efficacy | Flu/Cy /others | 1–5×10^6^/Kg | 80.9% CR/ CRi | 23.4% CRS  6.4% ICANS | OS and DFS  53% and 45%  respectively at 1y |
| (68) | 2020 | Sheba Medical Center | NCT00287131 | CD19 | 2G: CD28 | B-ALL  B-NHL | 90 | Flu/Cy | 1×10^6^/Kg | ALL: 84.4% CR (67% MRD neg);  NHL: 62% ORR and 31% CR | N/A | N/A |
| (69) | 2020 | Lyon Sud Hospital | N/A | CD19 | 2G: CD28  (Yescarta; Axicabta-gene Ciloleucel)  2G: 4-1BB  (Kymriah; Tisagen-lecleucel) | B-DLBCL | 61  Kymriah 33  Yescarta 28 | Flu/Cy (98%) Bendamustine (2%) | As per EMA approval | At 3m  Yescarta: 40% CR  8% PR  Kymriah  39% CR  3% PR | 8% CRS  10% ICANS | At med follow-up 5.7m    med PFS 3m; med OS 11.8m |
| (70) | 2020 | Multicentre China | NCT03097770 | CD19 & CD20 | 2G: 41BB  Bispecific CAR | B-NHL | 28 | Flu/Cy | 0.5-6×10^6^/Kg | 79% ORR  71% CR | 14% CRS  0% ICANS | PFS 64% at 12m |
| (71) | 2020 | Tongji Medical College | ChiCTR-OPN-16008526 | CD19 & CD22 | 3G: CD28 & 41BB sequential CAR T-cell infusions | B-ALL 51  B-NHL 38 | 89 | Flu/Cy | B-ALL 2.6×10^6^/Kg CAR19 & 2.7×10^6^/Kg CAR22  B-NHL  5.1×10^6^/Kg CAR19 &  5.3×10^6^/Kg CAR22 | B-ALL  96% CR  B-NHL 50% CR | 23.52% CRS  0% ICANS | B-ALL  med OS and PFS 31 and 13.6m respectively  B-NHL  med OS and PFS  18 and 9.9m respectively |
| (72) | 2020 | US Lymphoma CAR T Consortium | Axicabtagene Ciloleucel as standard of care | CD19 | 2G: CD28 | LBCL | 275 | Flu/Cy | As per FDA approval | 82% ORR  64% CR | 7% CRS  31% ICANS | at med follow-up 12.9m  med OS not reached  med PFS 8.3m |
| (73) | 2020 | Xuzhou Medical University | NCT03207178 | CD19 & CD20 | 2G: 41BB co-infusion of CAR T-cells | DLBCL | 21 | Flu/Cy (19) or ifosfamide (2) | CD19 CAR T-cell dose 0.2-4x10^6^/Kg  CD20 CAR T-cell dose 0.1-4.0x10^6^/Kg | 81% ORR  52.4% CR | 28.5% CRS  9.5% ICANS | med PFS 5.0m  med OS 8.1m |
| (74) | 2020 | Juno | NCT02631044  (TRANSCEND NHL-001) | CD19 | 2G: 4-1BB (Lisocabt-agene ma- raleucel) | B-NHL | 269 treated,  256 evaluable for efficacy | Flu/Cy | 50x10^6^  100x10^6^  150x10^6^ cells  1:1 CD4:CD8 | 73% ORR  53% CR  20% PR  13% SD | 2% CRS  10% ICANS | 44% PFS and 58% OS at 1y  med OS 21.1m |
| (75) | 2020 | Kite/ Gilead | NCT03105336  (ZUMA-5) | CD19 | 2G: CD28  (Yescarta; Axicabta-gene Ciloleucel) | Indolent NHL  (FL & MZL) | 146 treated (124 FL; 22MZL)  104 evaluable for efficacy | Flu/Cy | 2x10^6^/Kg | 92% ORR  76% CR | 7% CRS  19% ICANS | Med follow up 17.5m  92% ORR  76% CR |
| (76) | 2020 | First Affiliated Hospital of Zhengzhou University | N/A (Abstract only available) | CD19 | 2G: 41BB | B-NHL | 14 | N/A (Abstract only available) | N/A (Abstract only available) | 77% ORR  43% CR | 28.6% CRS | N/A (Abstract only available) |
| (77, 78) | 2020 | Kite/ Gilead | NCT02926833  (ZUMA-6) | CD19 | 2G: CD28 | DLBCL | 28 | Flu/Cy + atezolizumab | 2x10^6^/Kg | 75% ORR  46% CR | 4% CRS  29% ICANS | Med follow up 10.2m, 46% ongoing responders |
| (79) | 2020 | Autolus | NCT04404660  (ALLCAR) | CD19 | 2G: 41BB  AUTO1 | B-ALL | 20 treated  19 evaluable for efficacy | Flu/Cy | 410 x 10^6^ cells as split dose d1 and 10 | 84% CR | 0% CRS  15% ICANS | EFS at 6m 69% EFS at 12m 52% |
| (80) | 2020 | Kite/ Gilead | NCT03761056  (ZUMA-12) | CD19 | 2G: CD28  (Yescarta; Axicabta-gene Ciloleucel) | High risk  LBCL | 31  12 evaluable for efficacy  15 evaluable for safety | Flu/Cy | 2x10^6^/Kg | 92% ORR  75% CR | 20% CRS  27% ICANS | 75% CRs persisted at data cut-off |
| ***Other haematological malignancies*** | | | | | | | | | | | | |
| (9) | 2016 | Baylor | NCT00881920 | κ light chain | 2G: CD28 | MM | 7 | Cy unless lymphopenic | 1.7x10^7^ to 1.9x10^8^/m^2^ | 4 SD | 0% CRS  0% ICANS | SD for 117m and 24m |
| (81) | 2016 | NIH | NCT02215967 | BCMA | 2G: CD28 | MM | 12 | Flu/Cy | 0.3x10^6^  to 9x10^6^ /Kg | 8% CR  8% PR (both at highest dose level)  67% SD | 33% CRS  8% ICANS (?grades) | 8% ongoing response at 26w |
| (82) | 2017 | Chinese PLA General Hospital | NCT02259556 | CD30 | 2G: 41BB | HD | 18 | Flu/Cy or two others | 1x10^7^ to  3x10^7^/Kg | 39% PR | 0% CRS  0% ICANS | med PFS  6 mo |
| (83) | 2017 | Baylor | NCT01316146 | CD30 | 2G: CD28 | HD/ALCL | 9 | Nil | 2x10^7^/m^2^  1x10^8^/m^2^  2x10^8^/m^2^ | 33% CR | 0% CRS  0% ICANS | 2 CRs for 9 & 30 months |
| (84) | 2018 | Juno | NCT03430011  (EVOLVE) | BCMA | 2G: 41BB | MM | 13 treated  8 evaluable for efficacy | Flu/Cy | 50x10^6^  150x10^6^ cells | 100% ORR  38% CR | 0% CRS  12% ICANS | At data cut, no patients had progressed |
| (85) | 2018 | MSKCC | NCT03070327 | BCMA | 2G: 41BB | MM | 11 | Cy or Flu/Cy | 72-818x10^6^ cells | 64% ORR | 20% CRS  0% ICANS | med duration response 106d |
| (86) | 2018 | UPenn | NCT02135406 | CD19 | 2G: 41BB | MM | 10 | High dose melphalan and auto HSCT | 1×10^7^ to 5×10^7^ | 80% ORR | 0% CRS  0% ICANS | med PFS 200d |
| (87) | 2018 | Celyad Oncology | NCT03018405  (Think study) | NKG2D ligands | 1G plus endog. DAP10  (CYAD-01) | AML  MDS  MM | 16 treated  13 evaluable for efficacy | Nil | 3x10^8^  1x10^9^  3x10^9^  (multiple infusions) | 46% ORR (3 CRi/h in AML cohort) | 42% CRS  0% ICANS | One AML CR patient bridged to allo HSCT and in CR for >1y |
| (88) | 2018 | The Second Affiliated Hospital of Henan University of Traditional Chinese Medicine | NCT03093168 | BCMA | 2G: 41BB | MM | 17 treated  14 evaluable for efficacy | Flu/Cy | 9x10^6^ /Kg | 79% ORR  50% CR | 7% CRS  7% ICANS | Two responses sustained at 15m |
| (89) | 2018 | FHCRC | NCT03338972 | BCMA | 2G: 41BB | MM | 7 | Flu/Cy | 5 to 15×10^7^ cells  1:1 CD4:CD8 | 100% ORR | 0% CRS  0% ICANS | All surviving at a median of 16w follow up |
| (90) | 2018 | Huazhong University of Science and Technology | ChiCTR-OPC-16009113 | BCMA | 2G: CD28 | MM | 28 | Flu/Cy | 5.4-25×10^6^/Kg | 93% ORR  If BCMA >50%  CR 73%  If BCMA  <50% CR & VGPR 33% | 14% CRS | Med PFS 296d and 64d  based on BCMA expression >50% and <50% respectively |
| (91) | 2018 | Jiangsu Institute of Hematology | NCT03455972 | CD19 & BCMA | 3G: CD28 + OX40  Sequential CAR T-cell infusions | MM | 9 | Cy/Bu and auto HSCT | 1x10^6^/Kg CD19  1x10^6^/Kg BCMA | 100% ORR 33% CR | 0% CRS  0% ICANS | Not available as pts had further treatments |
| (92) | 2019 | Celgene/ Bluebird Bio | NCT02658929 | BCMA | 2G: 41BB  (BB2121; idecabta-gene  vicleucel) | MM | 33 | Flu/Cy | 50x10^6^ to  800x10^6^ cells | 85% ORR  45% CR | 6% CRS  3% ICANS | Med PFS 11.8m |
| (93) | 2019 | Xuzhou Medical University | ChiCTR-OIC-17011272 | CD19 & BCMA | Both 2G: 41BB and infused on the same day | MM | 21 | Flu/Cy | 1x10^6^ /Kg CD19 +  1x10^6^ /Kg BCMA | 95% ORR  57% CR | 4% CRS  0% ICANS | Med PFS for responders 243d |
| (94) | 2019 | Celyad Oncology | NCT02203825 | NKG2D ligands | 1G plus endog. DAP10  (CYAD-01 | AML  MM | 7 AML  5 MM | Nil | 1x10^6^  to 3x10^7^ cells | No responses | 0% CRS  0% ICANS | Med OS 4.7m |
| (95) | 2019 | Celyad Oncology | NCT03466320  (Deplethink) | NKG2D ligands | 1G plus endog. DAP10  (CYAD-01) | AML  MDS | 9 | Flu/Cy | 1x10^8^  3x10^8^  1x10^9^ cells | 0% ORR first 2 dose levels | 22% CRS  11% ICANS | 33% did not progress after 1m |
| (96) | 2019 | Tongji Medical College | ChiCTR1800018143 | BCMA& CD38 | 2G: 41BB  Bispecific CAR | MM | 16 | Flu/Cy | 0.5-4×10^6^/Kg | 85% ORR  50% sCR  12.5% VGPR  25% PR | 25% CRS  0% ICANS | Med duration of PFS not reached  75% PFS at 9m |
| (97) | 2019 | UPenn | NCT02546167 | BCMA | 2G: 41BB | MM | 25 | Cy  (cohorts 2&3) | 1×10^8^ to 5×10^8^  1×10^7^ to 5×10^7^  1×10^8^ to 5×10^8^ cells | 48% ORR  8% CR | 32% CRS  12% ICANS | Med duration of response 124.5d |
| (98) | 2019 | Tongji Medical College | ChiCTR1800018137 | BCMA | 2G: 41BB | MM | 9 | Flu/Cy | 1×10^6^ to 6×10^6^/Kg x 3 doses | 100% ORR  44% CR | 0% CRS  1 DLT at highest dose level | N/A |
| (99-101) | 2019 | Nanjing Legend Biotech Co | NCT03090659 | BCMA | 2G: 41BB  LCAR-B38M; JNJ-68284528; Ciltacabta-gene  autoleucel) | MM | 57 | Cy or Flu/Cy | 0.2 to 1.5 × 10^6^/Kg | 88% ORR  82% CR  6% PR | 41% CRS (1 fatal)  0% ICANS | Med OS not reached  Med PFS 12m |
| (102) | 2019 | The First Affiliated Hospital of Soochow University | NCT03196414 | CD19 & BCMA | 3G: CD28 + OX40  Sequential infusions | MM | 28 | Flu/Cy | CART-19 1×10^7^/kg  CART-BCMA 2-6.8×10^7^/kg | 92.6% ORR  40.7%CR | 32% CRS  3% ICANS | Med PFS 8m  Med OS 16m |
| (103) | 2019 | FHCRC | NCT03502577 | BCMA | 2G: 41BB | MM | 7 treated  6 analysed for efficacy | Flu/Cy + JSMD194  (γ-secretase inhibitor) | 5x10^7^ cells  1:1 CD4:CD8 | 100%ORR | 16% CRS (fatal)  70% ICANS | No relapses at median follow-up 5 months |
| (104) | 2019 | Multicentre China | NCT03716856  NCT03302403  NCT03380039 | BCMA | 2G: 41BB | MM | 24 | Flu/Cy | 0.5-1.8x10^8^ cells | 87.5% ORR  79.2% CR | 33% CRS  12.5% ICANS | 54% ongoing CR at med follow-up of 383d |
| (105) | 2019 | UCL | NCT03287804 | BCMA & TACI | 3G: CD28 + OX40 dual antigen targeted CAR  (AUTO2) | MM | 12 | Flu/Cy | 15 to 900x10^6^ cells | 43% ORR  28% PR | 0% CRS  0% ICANS | N/A |
| (106) | 2019 | NCI | NCT03602612 | BCMA | 2G: 41BB | MM | 12 | Flu/Cy | 0.75x10^6^/Kg  1.5x10^6^Kg  3x10^6^/Kg | 83% ORR  41% CR & VGPR | 8% CRS  8% ICANS | Best duration of response 2-37w |
| (107) | 2019 | Bluebird Bio | NCT03274219 | BCMA | 2G: 41BB  (bb21217; includes the PI3K inhibitor bb007* during culture) | MM | 22 treated  18 analysed | Flu/Cy | 150, 450, 800, 1200x10^6^ cells | 83% ORR | 5% CRS  9% ICANS | 50% ongoing response at >2m follow-up  (including 2 pts at 15&18m) |
| (108) | 2020 | Poseida Therapeutics | NCT03288493 | BCMA | 2G: 41BB | MM | 43 treated | Flu/Cy | 0.75-15× 10^6^ cells/Kg  Piggy/Bac transposon | 57% ORR | 3% CRS  0% ICANS | Median OS not reached |
| (109, 110) | 2020 | Celyad  Oncology | NCT04167696  (CYCLE-1) | NKG2D ligands | 1G plus endog. DAP10  (CYAD-02; includes shRNA targeting MICA/B) | AML  MDS | 9  treated  7 evaluable for efficacy | Flu/Cy | 1x10^8^  3x10^8^  1x10^9^ cells | 14% CR | 14% CRS  0% ICANS | Ongoing responses  at 4 & 6m |
| (111) | 2020 | CARsgen Therapeutics | NCT03975907  (Lummicar-1) | BCMA | 2G: 41BB | MM | 14 treated  12 evaluable for efficacy | Flu/Cy | 1.0-1.5×10^8^  CAR+ T-cells | 100% ORR  42% CR | 0% CRS  0% ICANS | N/A |
| (112) | 2020 | CARsgen Therapeutics | NCT03915184  (Lummicar-2) | BCMA | 2G: 41BB | MM | 14 treated  10 evaluable for efficacy | Flu/Cy | 0.5–1.8×10^8^ | 100% ORR  40% CR | 0% CRS  0% ICANS | N/A |
| (113) | 2020 | Celgene | NCT03361748  (KarMMa) | BCMA | 2G: 41BB  (BB2121; idecabta-gene vicleucel) | MM | 128 | Flu/Cy | 150×10^6^ to 450×10^6^ CAR+ T-cells | 73% ORR  31% CR | 5.5% CRS (1 fatal)  3% ICANS | Med PFS 8.6m  Med duration of response 10.6m  Med OS 19.4m |
| (114, 115) | 2020 | Janssen Research & Dvpt | NCT03548207  CARTITUDE-1 | BCMA | 2G: 41BB  (JNJ-68284528; LCAR-B38M; Ciltacabta-gene  autoleucel) | MM | 97 | Flu/Cy | Target dose 0.75 x10^6^/Kg (range 0.5-1.0x10^6^/Kg) | 94.8% ORR  55.7% sCR | 4.1% CRS (1 fatal)  10.3% ICANS (1 fatal) | 87.4% PFS at 6m  93.8% OS at 6m  Med duration of response not reached (med follow up 8.8m) |
| ***Solid Tumours*** | | | | | | | | | | | | |
| (116-118) | 1998  2002 | Cell Genesys | N/A | TAG-72 | 1G | Colorectal cancer | 16 | Nil | Up to 10^10^ cells by IV (10) or intrahepatic artery infusion (6) | No responses | Hyperbilirubin-aemia n=2 | N/A |
| (117) | 2002 | N/A | N/A | CEA | 1G | Colorectal cancer and breast cancer | 7 | Nil | Up to 10^11^ cells + IL-2 (n=2) | Two “minor responses” | Tolerance “adequate” | N/A |
| (119) | 2006 | NCI | N/A | FR-α | 1G | Ovarian cancer | 14 | Nil | 3-216x10^9^ in 1-2 doses + IL-2 (n=8) | No responses | Toxicity attributed to IL-2 | N/A |
| (120) | 2007 | Children’s Hospital Seattle | N/A | L1-CAM | 1G | Neurobl-astoma | 6 | Nil | 1-11x10^8^/m^2^ in 1-2 doses + IL-2 in some cases | Cannot assess owing to additional therapies | 0% CRS  0% ICANS | Died of disease d162-1670 |
| (121-123) | 2006-2013 | Erasmus University Medical Center | N/A | CAIX | 1G | Renal cell carcinoma | 12 | Nil | 0.2-2.1x10^9^ cells | No responses | 33% hepatotoxicity due to on-target off-tumour toxicity | Med OS 9.5m (12.5m in 4 patients who received a CAIX blocking antibody prior to CAR T-cells) |
| (124, 125) | 2008  2011 | Baylor | NCT00085930 | GD2 | 1G | Neurobl-astoma | 19  Treated  11 evaluable for efficacy | Nil | 2-20x10^7^ cells | 27% CR  9% PR | 0% CRS  0% ICANS | Two CR durable for >60m and >21m |
| (126) | 2013 | MSKCC | NCT01140373 | PSMA | 2G: CD28 | Prostate cancer | 7 | Cy | 1 to 3 x10^7^/kg | 29% SD | Pyrexia up to 39°C | SD for 6 and 16m |
| (127) | 2015 | Baylor | NCT00902044 | HER2 | 2G: CD28 | Sarcoma | 19 | Nil | 1x10^4^ to 1x10^8^cells/m^2^ | 24% SD for 12w to 14m | 0% CRS 0 % ICANS | Med OS 10.3m |
| (128) | 2015 | Roger Williams Medical Center | NCT01373047 | CEA | 2G: CD28 | CEA+ metastatic liver disease | 6 | Nil | 1x10^8^  to 1x10^10^ cells by 3 sequential intrahepatic artery infusions + IL-2 in 3 cases | 17% SD | 0% CRS 0 % ICANS | Med OS 15w |
| (129) | 2016 | Chinese PLA General Hospital | NCT01869166 | EGFR | 2G: 41BB | Non-small cell lung cancer | 11 | Nil, Cy alone or Cy with additional cytotoxic drugs | Median dose 0.97x10^7^/kg | 18% PR  45% SD | 0% CRS 0% ICANS | Responses lasted 2-8m |
| (130) | 2017 | Chinese PLA General Hospital | NCT01869166 | EGFR | 2G: 41BB | Biliary tract cancer | 19 treated  17 analysed for efficacy | Cy/ nab-paclitaxel | Median 2.65 x10^6^/kg x 1-3 cycles | 6% CR  59% SD | 15% CRS (grade 3 acute fever/ chill)  0% ICANS | Med PFS 4m |
| (131) | 2017 | The Christie NHS | NCT01212887 | CEA | 1G | CEA+ malignancy | 14 | Fludarabine alone or Flu/Cy + IL-2 | Up to 5x10^10^ cells | No responses  50% SD | On-target off-tumour pulmonary toxicity | No long term sustained  responses |
| (132) | 2017 | Third Military Medical University | NCT02349724 | CEA | 2G: CD28 | Colorectal cancer | 10 | Cy | 1 x10^5^ to 1 x10^8^/kg | 70% SD | 0% CRS 0 % ICANS | 20% SD at 30w follow up |
| (133) | 2017 | UPenn | NCT01837602 | MET | 2G: 41BB | Breast cancer | 6 | Nil | 3x10^7^  3x10^8^ cells  mRNA transfected CAR T-cells administered directly to tumour | No responses | 0% CRS  0% ICANS | 1SD at med follow up 10m |
| (134) | 2017 | Baylor | NCT01822652 | GD2 | 3G: CD28/  OX40 | Neurobl-astoma | 11 | Nil or Flu/Cy  Final cohort received Flu/Cy+PD-1 inhibitor | 1-17x10^7^/m^2^ | 45% SD | 0% CRS  0% ICANS | Med OS 506d |
| (135) | 2017 | UPenn | NCT02209376 | EGFR  vIII | 2G: 41BB | GBM | 10 | Nil | 1x10^8^  to 5x10^8^ CAR+ cells | 90% SD  (Antigen loss noted in 5/7 tumours) | 0% CRS  ICANS not easily assessed due to tumour location | Med OS 8m |
| (136) | 2017 | Baylor | NCT01109095 | HER2 | 2G: CD28 | GBM  Adults/  children | 17 | Nil | 1x10^6^  to 1x10^8^ /m^2^  virus-specific T-cells (CMV, EBV, adenovirus) | 6% PR  41% SD | 0% CRS 0% ICANS | Med OS 11.1m |
| (137) | 2018 | Chinese PLA General Hospital | NCT01935843 | HER2 | 2G: 41BB | Biliary tract and pancreatic cancer | 11 | Cy/ nab-paclitaxel | Median dose  2.1x10^6^/Kg x 1-2 cycles | 9% PR  45% SD | 0% CRS  0% ICANS | Med PFS 4.8m |
| (138) | 2018 | UPenn | NCT01897415 | Meso-thelin | 2G: 41BB | Pancreatic cancer | 6 | Nil | mRNA transfected CAR T-cells x 9 infusions of 1-3x10^8^/m^2^  cells over 3 weeks | 33% SD  Reduction in FDG uptake seen by PET in one patient | 0% CRS  0% ICANS | PFS 3.8 and 5.4m in 2 patients |
| (139) | 2018 | King’s College London | NCT01818323 | ErbB family | 2G: CD28 | Squamous cell carcinoma of head and neck | 13 | Nil | 1x10^7^ to  1x10^9^ CAR+ cells by intra-tumoural injection | 69% SD | 0% CRS  0% ICANS | N/A |
| (140) | 2019 | UPenn | NCT02159716 | Meso-thelin | 2G: 41BB | Malignant pleural mesothel-ioma  Ovarian cancer  Pancreatic cancer | 15 | Nil or Cy | 1-3x10^7^or  1-3x10^8^/m^2^ cells | 73% SD | 0% CRS  0% ICANS | Med PFS 2.1m |
| (141) | 2019 | Bellicum Pharma-ceuticals | NCT02744287 | PSCA | 1G + rimiducid-inducible MyD88/  CD40 co-activation switch  (BPX-601) | PSCA-expressing cancers | 15 | Nil, Cy or Flu/Cy | 1.25x10^6^  1.25x10^6^ +rimiducid  2.25x10^6^ +rimiducid | 53% SD | 0% CRS  0% ICANS | N/A |
| (142, 143) | 2019 | Baylor | NCT00902044 | HER2 | 2G: CD28 | Sarcomas  (paediatric) | 10 | Fludarabine alone or Flu/Cy | 1x10^8^/m^2^  Up to 3 infusions post LD followed, in some cases, by further infusions without LD | 20% CR | 0% CRS  0% ICANS | Two survivors at 32 and 33m |
| (144, 145) | 2019 | UPenn | NCT03089203 | PSMA | 2G: 41BB  + TGF-β dominant negative receptor | Prostate | 10 | Nil or Flu/Cy (cohort 3) | 1-3x10^7^/m^2^  1-3x10^8^/m^2^  1-3x10^8^/m^2^ cells | Med 33.2%  PSA decline | 40% CRS  DLT cohort 3 | N/A |
| (146) | 2019 | Changhai Hospital | NCT03159819 | Claudin 18.2 | 2G: CD28 | Gastric or pancreatic cancer | 12  11 evaluable for efficacy | Flu/Cy alone or with nab-paclitaxel | 0.5-55x10^8^ CAR+ cells administered over 1-5 cycles | 9% CR  27% PR  45% SD | 0% CRS  0% ICANS | Med PFS 130d |
| (147) | 2019 | MSKCC | NCT02414269 | Meso-thelin | 2G: CD28 | Pleural cancers | 20  14 in cohort 3 evaluable for efficacy | Nil (3)  Cy (3)  Cy + anti-PD-1 (14) | 3x10^5^-1x10^7^/Kg | 14% CR  36% PR  29% SD | 0% CRS  0% ICANS | N/A |
| (148) | 2019 | NCI | NCT01454596 | EGFR  vIII | 3G: CD28 & 41BB | GBM | 18 | Flu/Cy + IL-2 | 6.3 x10^6^ to 2.6x10^10^ CAR+ cells | No responders | 1 fatal pulmonary event event at highest dose level (?CRS)  1 grade 3 neurological event | Med PFS 1.3m  Med OS 6.9m |
| (149) | 2019 | Beijing Sanbo Brain Hospital | NCT02937844 | PD-L1/2 | PD1-CD28 switch receptor | GBM | 14 | Cy | Up to 1x10^9^ cells over three IV infusions. One patient received IV & intracavitary cells | Transient response | 0% CRS  0% ICANS | Med OS 4.4 months |
| (150) | 2020 | Roger Williams  Medical Centre | NCT02416466  (HITM-SIR) | CEA | 2G: CD28 | CEA+ metastatic liver disease | 6 | Selective intra-arterial radiation with SIR spheres | 1x10^10^ cells by 3 sequential intrahepatic artery infusions & IL-2 | 16% SD prior to SIR sphere administration | 0% CRS 0 % ICANS | Med OS 8m |
| (151) | 2020 | RenJi Hospital | NCT02395250 NCT03146234 | GLP-3 | 2G: CD28 | HCC | 13 | Cy alone or Flu/Cy | 7-40.77x10^8^ CAR+ cells administered over 2-9 cycles | 15% PR | 8% CRS (case proved fatal)  0% ICANS | OS at 3y 10.5% |
| (152) | 2020 | UCL/ Cancer Research UK | [NCT02761915](http://clinicaltrials.gov/show/NCT02761915) | GD2 | 2G: CD28  (AUTO6) | Neurobl-astoma | 12 | Nil  Cy  Flu/Cy | 1x10^7^-1x10^9^ CAR+ T-cells/m^2^ | No responses but disease regression noted in 3 patients | 8% CRS | Patients with disease regression died on d50 and within 5m (n=2) |
| (153) | 2020 | MSKCC | NCT02498912 | MUC16 | 2G: CD28 + IL-12 | Ovarian cancer | 16 | Nil or Flu/Cy | 3x10^5^-1x10^7^/Kg | No responses (best response stable disease) | 2/3 developed macrophage activation-like syndrome in Flu/Cy cohort | N/A |
| ***Allogeneic CAR T-cells and NK cells*** | | | | | | | | | | | | |
| (154) | 2013 | Baylor | NCT00840853 | CD19 | 2G: CD28 | B-ALL  B-CLL | 8 treated  6 evaluable | 3m-13y post allo HSCT  No conditioning | 1.5x10^7^/m^2^  4.5x10^7^/m^2^  1.2x10^8^/m^2^  donor-derived virus-specific T-cells | 38% CR (2/3 patients in CR at time of treatment)  12% PR | 0% CRS  0% ICANS  0% GvHD | Follow-up still ongoing in 2 CR patients at 2 and 8 months. Third CR lasted 3m |
| (155) (156) | 2013  2016 | NCI | NCT01087294 | CD19 | 2G: CD28 | B-cell malignancy relapsed post allo HSCT | 20 | No conditioning | 1x10^6^ to  10x10^6^/Kg  donor derived T-cells | 30% CR  10% PR | 50% CRS (inferred from information provided)  0% ICANS  0% GvHD | CR sustained for 3-30m at time of writing |
| (16) | 2016 | MDACC | NCT01497184 | CD19 | 2G: CD28 | B-ALL  B-NHL | 19 | Allogeneic HSCT | 10^6^ to 10^8^/m^2^  Sleeping beauty transposon | 58% CR | 0% CRS  0% ICANS  No exacerbation of GvHD | 53% PFS at 12 mo |
| (157) | 2017 | Multicentre UK & Germany | NCT01195480 | CD19 | 1G | B-ALL (paediatric) relapsed post HSCT or at high risk of relapse post second allo HSCT | 6 | Fludarabine | 4x10^7^-2x10^8^/m^2^  donor-derived EBV-specific T-cells | 33% CR (2/2 were in CR at time of infusion)  16% PR | 0% CRS  0% ICANS  0% GvHD | One survivor at 3y |
| (157) | 2017 | Multicentre UK & Germany | NCT01195480 | CD19 | 1G | B-ALL (paediatric) relapsed post HSCT or at high risk of relapse post second allo HSCT | 6 | Fludarabine + vaccination with EBV transformed lympho-blastoid cell line | 2x10^8^/m^2^  donor-derived EBV-specific T-cells | 50% CR (2/3 were in CR at time of infusion) | 0% CRS  0% ICANS  0% GvHD | All patients relapsed |
| (158)  (159) | 2019 | Celyad Oncology | NCT03692429  ALLOSHRINK | NKG2D ligands | 1G plus endog. DAP10  (CYAD-101, also comprising truncated CD3ζ to reduce  ­­­activation via endog. TCR) | CRC | 15 | Concurrent FOLFOX chemotherapy x 3 cycles | 1x10^8^  3x10^8^  1x10^9^ cells  x 3 infusions with concurrent FOLFOX | 13% PR  60% SD | 0% CRS  0% ICANS  0% GvHD | Med OS 10.6m  Med PFS 3.9m |
| (160)  (161) | 2020 | Allogene  Therapeutics | NCT03939026  (ALPHA) | CD19 | 2G: 41BB  (ALLO-501) | B-NHL | 22 treated  19 evaluable (efficacy) | Flu/Cy/ Allo-647 (two dose levels Allo-647) | 40x10^6^  160x10^6^  360x10^6^ cells  TALEN disruption of TRAC and CD52 | 63% ORR  37% CR  26% PR | 5% CRS  0% ICANS  0% GvHD | 9 of 12 patients remain in response at med follow up of 3.8m |
| (162) (163) | 2020 | Allogene  Therapeutics | NCT04093596  (UNIVERSAL) | BCMA | 2G: 41BB  (ALLO-715) | MM | 31 treated  26 evaluable (efficacy) | Flu/Cy/ Allo-647 (two dose levels Allo-647) or Cy/Allo-647 | 40x10^6^  160x10^6^  320x10^6^  480x10^6^ cells  TALEN disruption of TRAC and CD52 | At dose level 3  60% ORR  40% CR or VGPR | 0% CRS  0% ICANS  0% GvHD | Med follow-up 3.2m  6 /9 treated at DL3 or DL4 still in response |
| (164) | 2020 | CRISPR Therapeutics | NCT04035434  (CARBON) | CD19 | 2G: CD28  (CTX110) | B-NHL | 11 | Flu/Cy | 30x10^6^  100x10^6^  300x10^6^  600x10^6^ cells  CRISPR knock-in at TRAC locus with β2M knockout | 36% CR | 30% CRS  10% ICANS  0% GvHD | Death of only patient treated at highest dose level  (herpes viral reactivation)  CR still ongoing at 1 month |
| (165) | 2020 | MDACC | NCT03056339 | CD19 | 2G: CD28  + IL-15 + inducible caspase 9 suicide gene | B-NHL  B-CLL | 11 | Flu/Cy | 1x10^5^ to  1x10^7^/Kg  Umbilical cord blood derived NK cells | 64% CR  9% PR | 0% CRS  0% ICANS  0% GvHD | Other therapies administered >30d after CAR-NK |
| (166) | 2020 | Multicentre | NCT02808442 &  NCT02746952  (UCART19) | CD19 | 2G: 41BB | B-ALL  (7 pediatric,  14 adults) | 21 | Flu/Cy with or without alemtuzumab | 1·1-2·3 × 10^6^ kg (paediatrics)  6×10^6^, 6-8×10^7^ or 1·8-2·4×10^8^ cells dose-escalation (adults)  TALEN knockout of TRAC and CD52 | 67% CR or CRi | 14% CRS  0% ICANS | 55% OS  27% PFS at 6m |

**References**

1. Brentjens RJ, Riviere I, Park JH, Davila ML, Wang X, Stefanski J, et al. Safety and persistence of adoptively transferred autologous CD19-targeted T cells in patients with relapsed or chemotherapy refractory B-cell leukemias. Blood. 2011;118(18):4817-28.

2. Savoldo B, Ramos CA, Liu E, Mims MP, Keating MJ, Carrum G, et al. CD28 costimulation improves expansion and persistence of chimeric antigen receptor-modified T cells in lymphoma patients. J Clin Invest. 2011;121(5):1822-6.

3. Kochenderfer JN, Dudley ME, Feldman SA, Wilson WH, Spaner DE, Maric I, et al. B-cell depletion and remissions of malignancy along with cytokine-associated toxicity in a clinical trial of anti-CD19 chimeric-antigen-receptor-transduced T cells. Blood. 2012;119(12):2709-20.

4. Davila ML, Riviere I, Wang X, Bartido S, Park J, Curran K, et al. Efficacy and toxicity management of 19-28z CAR T cell therapy in B cell acute lymphoblastic leukemia. Sci Transl Med. 2014;6(224):224ra25.

5. Maude SL, Frey N, Shaw PA, Aplenc R, Barrett DM, Bunin NJ, et al. Chimeric antigen receptor T cells for sustained remissions in leukemia. N Engl J Med. 2014;371(16):1507-17.

6. Porter DL, Hwang WT, Frey NV, Lacey SF, Shaw PA, Loren AW, et al. Chimeric antigen receptor T cells persist and induce sustained remissions in relapsed refractory chronic lymphocytic leukemia. Sci Transl Med. 2015;7(303):303ra139.

7. Kochenderfer JN, Dudley ME, Kassim SH, Somerville RP, Carpenter RO, Stetler-Stevenson M, et al. Chemotherapy-refractory diffuse large B-cell lymphoma and indolent B-cell malignancies can be effectively treated with autologous T cells expressing an anti-CD19 chimeric antigen receptor. J Clin Oncol. 2015;33(6):540-9.

8. Lee DW, Kochenderfer JN, Stetler-Stevenson M, Cui YK, Delbrook C, Feldman SA, et al. T cells expressing CD19 chimeric antigen receptors for acute lymphoblastic leukaemia in children and young adults: a phase 1 dose-escalation trial. Lancet. 2015;385(9967):517-28.

9. Ramos CA, Savoldo B, Torrano V, Ballard B, Zhang H, Dakhova O, et al. Clinical responses with T lymphocytes targeting malignancy-associated κ light chains. J Clin Invest. 2016;126(7):2588-96.

10. Zhang WY, Wang Y, Guo YL, Dai HR, Yang QM, Zhang YJ, et al. Treatment of CD20-directed Chimeric Antigen Receptor-modified T cells in patients with relapsed or refractory B-cell non-Hodgkin lymphoma: an early phase IIa trial report. Signal Transduct Target Ther. 2016;1:16002.

11. Porter DL, Frey NV, Melenhorst JJ, Hwang W-T, Lacey SF, Shaw PA, et al. Randomized, phase II dose optimization study of chimeric antigen receptor (CAR) modified T cells directed against CD19 in patients (pts) with relapsed, refractory (R/R) CLL. Journal of Clinical Oncology. 2016;34(15_suppl):3009-.

12. Maude SL, Teachey DT, Rheingold SR, Shaw PA, Aplenc R, Barrett DM, et al. Sustained remissions with CD19-specific chimeric antigen receptor (CAR)-modified T cells in children with relapsed/refractory ALL. Journal of Clinical Oncology. 2016;34(15_suppl):3011-.

13. Wang X, Popplewell LL, Wagner JR, Naranjo A, Blanchard MS, Mott MR, et al. Phase 1 studies of central memory-derived CD19 CAR T-cell therapy following autologous HSCT in patients with B-cell NHL. Blood. 2016;127(24):2980-90.

14. Turtle CJ, Hanafi LA, Berger C, Gooley TA, Cherian S, Hudecek M, et al. CD19 CAR-T cells of defined CD4+:CD8+ composition in adult B cell ALL patients. J Clin Invest. 2016;126(6):2123-38.

15. Turtle CJ, Hanafi L-A, Berger C, Hudecek M, Pender B, Robinson E, et al. Immunotherapy of non-Hodgkin’s lymphoma with a defined ratio of CD8<sup>+</sup> and CD4<sup>+</sup> CD19-specific chimeric antigen receptor–modified T cells. Science Translational Medicine. 2016;8(355):355ra116-355ra116.

16. Kebriaei P, Singh H, Huls MH, Figliola MJ, Bassett R, Olivares S, et al. Phase I trials using Sleeping Beauty to generate CD19-specific CAR T cells. J Clin Invest. 2016;126(9):3363-76.

17. Gill S, Frey NV, Hexner EO, Lacey SF, Melenhorst JJ, Byrd JC, et al. CD19 CAR-T cells combined with ibrutinib to induce complete remission in CLL. Journal of Clinical Oncology. 2017;35(15_suppl):7509-.

18. Schuster SJ, Svoboda J, Chong EA, Nasta SD, Mato AR, Anak Ö, et al. Chimeric Antigen Receptor T Cells in Refractory B-Cell Lymphomas. N Engl J Med. 2017;377(26):2545-54.

19. Gardner RA, Finney O, Annesley C, Brakke H, Summers C, Leger K, et al. Intent-to-treat leukemia remission by CD19 CAR T cells of defined formulation and dose in children and young adults. Blood. 2017;129(25):3322-31.

20. Turtle CJ, Hay KA, Hanafi LA, Li D, Cherian S, Chen X, et al. Durable Molecular Remissions in Chronic Lymphocytic Leukemia Treated With CD19-Specific Chimeric Antigen Receptor-Modified T Cells After Failure of Ibrutinib. J Clin Oncol. 2017;35(26):3010-20.

21. Hu Y, Wu Z, Luo Y, Shi J, Yu J, Pu C, et al. Potent Anti-leukemia Activities of Chimeric Antigen Receptor–Modified T Cells against CD19 in Chinese Patients with Relapsed/Refractory Acute Lymphocytic Leukemia. Clinical Cancer Research. 2017;23(13):3297-306.

22. Kochenderfer JN, Somerville RPT, Lu T, Shi V, Bot A, Rossi J, et al. Lymphoma Remissions Caused by Anti-CD19 Chimeric Antigen Receptor T Cells Are Associated With High Serum Interleukin-15 Levels. J Clin Oncol. 2017;35(16):1803-13.

23. Pan J, Yang JF, Deng BP, Zhao XJ, Zhang X, Lin YH, et al. High efficacy and safety of low-dose CD19-directed CAR-T cell therapy in 51 refractory or relapsed B acute lymphoblastic leukemia patients. Leukemia. 2017;31(12):2587-93.

24. Park JH, Riviere I, Gonen M, Wang X, Senechal B, Curran KJ, et al. Long-Term Follow-up of CD19 CAR Therapy in Acute Lymphoblastic Leukemia. N Engl J Med. 2018;378(5):449-59.

25. Fry TJ, Shah NN, Orentas RJ, Stetler-Stevenson M, Yuan CM, Ramakrishna S, et al. CD22-targeted CAR T cells induce remission in B-ALL that is naive or resistant to CD19-targeted CAR immunotherapy. Nat Med. 2018;24(1):20-8.

26. Enblad G, Karlsson H, Gammelgård G, Wenthe J, Lövgren T, Amini RM, et al. A Phase I/IIa Trial Using CD19-Targeted Third-Generation CAR T Cells for Lymphoma and Leukemia. Clin Cancer Res. 2018;24(24):6185-94.

27. Jacoby E, Bielorai B, Avigdor A, Itzhaki O, Hutt D, Nussboim V, et al. Locally produced CD19 CAR T cells leading to clinical remissions in medullary and extramedullary relapsed acute lymphoblastic leukemia. Am J Hematol. 2018;93(12):1485-92.

28. Li S, Zhang J, Wang M, Fu G, Li Y, Pei L, et al. Treatment of acute lymphoblastic leukaemia with the second generation of CD19 CAR-T containing either CD28 or 4-1BB. Br J Haematol. 2018;181(3):360-71.

29. Cao J, Wang G, Cheng H, Wei C, Qi K, Sang W, et al. Potent anti-leukemia activities of humanized CD19-targeted Chimeric antigen receptor T (CAR-T) cells in patients with relapsed/refractory acute lymphoblastic leukemia. Am J Hematol. 2018;93(7):851-8.

30. Maude SL, Laetsch TW, Buechner J, Rives S, Boyer M, Bittencourt H, et al. Tisagenlecleucel in Children and Young Adults with B-Cell Lymphoblastic Leukemia. N Engl J Med. 2018;378(5):439-48.

31. Grupp SA, Maude SL, Rives S, Baruchel A, Boyer MW, Bittencourt H, et al. Updated Analysis of the Efficacy and Safety of Tisagenlecleucel in Pediatric and Young Adult Patients with Relapsed/Refractory (r/r) Acute Lymphoblastic Leukemia. Blood. 2018;132:895.

32. Cheng Z, Wei R, Ma Q, Shi L, He F, Shi Z, et al. In Vivo Expansion and Antitumor Activity of Coinfused CD28- and 4-1BB-Engineered CAR-T Cells in Patients with B Cell Leukemia. Mol Ther. 2018;26(4):976-85.

33. Schuster SJ, Bishop MR, Tam CS, Waller EK, Borchmann P, McGuirk JP, et al. Tisagenlecleucel in Adult Relapsed or Refractory Diffuse Large B-Cell Lymphoma. New England Journal of Medicine. 2018;380(1):45-56.

34. Geyer MB, Rivière I, Sénéchal B, Wang X, Wang Y, Purdon TJ, et al. Autologous CD19-Targeted CAR T Cells in Patients with Residual CLL following Initial Purine Analog-Based Therapy. Mol Ther. 2018;26(8):1896-905.

35. Hossain N, Sahaf B, Abramian M, Spiegel JY, Kong K, Kim S, et al. Phase I Experience with a Bi-Specific CAR Targeting CD19 and CD22 in Adults with B-Cell Malignancies. Blood. 2018;132(Supplement 1):490-.

36. Ying Z, Long L, Liu H, Song Y, Rizzieri DA, Nejadnik B, et al. ET190L1-ArtemisTM T Cell Therapy Results in Durable Disease Remissions with No Cytokine Release Syndrome or Neurotoxicity in Patients with Relapsed and Refractory B-Cell Lymphoma. Blood. 2018;132(Supplement 1):1689-.

37. Locke FL, Ghobadi A, Jacobson CA, Miklos DB, Lekakis LJ, Oluwole OO, et al. Long-term safety and activity of axicabtagene ciloleucel in refractory large B-cell lymphoma (ZUMA-1): a single-arm, multicentre, phase 1-2 trial. Lancet Oncol. 2019;20(1):31-42.

38. Sauter CS, Senechal B, Rivière I, Ni A, Bernal Y, Wang X, et al. CD19 CAR T cells following autologous transplantation in poor-risk relapsed and refractory B-cell non-Hodgkin lymphoma. Blood. 2019;134(7):626-35.

39. Hay KA, Gauthier J, Hirayama AV, Voutsinas JM, Wu Q, Li D, et al. Factors associated with durable EFS in adult B-cell ALL patients achieving MRD-negative CR after CD19 CAR T-cell therapy. Blood. 2019;133(15):1652-63.

40. Hirayama AV, Gauthier J, Hay KA, Voutsinas JM, Wu Q, Gooley T, et al. The response to lymphodepletion impacts PFS in patients with aggressive non-Hodgkin lymphoma treated with CD19 CAR T cells. Blood. 2019;133(17):1876-87.

41. Ghorashian S, Kramer AM, Onuoha S, Wright G, Bartram J, Richardson R, et al. Enhanced CAR T cell expansion and prolonged persistence in pediatric patients with ALL treated with a low-affinity CD19 CAR. Nat Med. 2019;25(9):1408-14.

42. Yan ZX, Li L, Wang W, OuYang BS, Cheng S, Wang L, et al. Clinical Efficacy and Tumor Microenvironment Influence in a Dose-Escalation Study of Anti-CD19 Chimeric Antigen Receptor T Cells in Refractory B-Cell Non-Hodgkin's Lymphoma. Clin Cancer Res. 2019;25(23):6995-7003.

43. Hirayama AV, Gauthier J, Hay KA, Voutsinas JM, Wu Q, Pender BS, et al. High rate of durable complete remission in follicular lymphoma after CD19 CAR-T cell immunotherapy. Blood. 2019;134(7):636-40.

44. Curran KJ, Margossian SP, Kernan NA, Silverman LB, Williams DA, Shukla N, et al. Toxicity and response after CD19-specific CAR T-cell therapy in pediatric/young adult relapsed/refractory B-ALL. Blood. 2019;134(26):2361-8.

45. Ma F, Ho JY, Du H, Xuan F, Wu X, Wang Q, et al. Evidence of long-lasting anti-CD19 activity of engrafted CD19 chimeric antigen receptor-modified T cells in a phase I study targeting pediatrics with acute lymphoblastic leukemia. Hematol Oncol. 2019;37(5):601-8.

46. Frigault MJ, Dietrich J, Martinez-Lage M, Leick M, Choi BD, DeFilipp Z, et al. Tisagenlecleucel CAR T-cell therapy in secondary CNS lymphoma. Blood. 2019;134(11):860-6.

47. Ying Z, Huang XF, Xiang X, Liu Y, Kang X, Song Y, et al. A safe and potent anti-CD19 CAR T cell therapy. Nat Med. 2019;25(6):947-53.

48. Jiang H, Li C, Yin P, Guo T, Liu L, Xia L, et al. Anti-CD19 chimeric antigen receptor-modified T-cell therapy bridging to allogeneic hematopoietic stem cell transplantation for relapsed/refractory B-cell acute lymphoblastic leukemia: An open-label pragmatic clinical trial. American Journal of Hematology. 2019;94(10):1113-22.

49. Cao Y, Lu W, Sun R, Jin X, Cheng L, He X, et al. Anti-CD19 Chimeric Antigen Receptor T Cells in Combination With Nivolumab Are Safe and Effective Against Relapsed/Refractory B-Cell Non-hodgkin Lymphoma. Frontiers in Oncology. 2019;9(767).

50. Ying Z, He T, Wang X, Zheng W, Lin N, Tu M, et al. Parallel Comparison of 4-1BB or CD28 Co-stimulated CD19-Targeted CAR-T Cells for B Cell Non-Hodgkin's Lymphoma. Mol Ther Oncolytics. 2019;15:60-8.

51. Geyer MB, Rivière I, Sénéchal B, Wang X, Wang Y, Purdon TJ, et al. Safety and tolerability of conditioning chemotherapy followed by CD19-targeted CAR T cells for relapsed/refractory CLL. JCI Insight. 2019;5(9).

52. Pan J, Niu Q, Deng B, Liu S, Wu T, Gao Z, et al. CD22 CAR T-cell therapy in refractory or relapsed B acute lymphoblastic leukemia. Leukemia. 2019;33(12):2854-66.

53. Tu S, Huang R, Guo Z, Deng L, Song C, Zhou X, et al. Shortening the ex vivo culture of CD19-specific CAR T-cells retains potent efficacy against acute lymphoblastic leukemia without CAR T-cell-related encephalopathy syndrome or severe cytokine release syndrome. Am J Hematol. 2019;94(12):E322-e5.

54. Amrolia PJ, Wynn R, Hough RE, Vora A, Bonney D, Veys P, et al. Phase I Study of AUTO3, a Bicistronic Chimeric Antigen Receptor (CAR) T-Cell Therapy Targeting CD19 and CD22, in Pediatric Patients with Relapsed/Refractory B-Cell Acute Lymphoblastic Leukemia (r/r B-ALL): Amelia Study. Blood. 2019;134(Supplement_1):2620-.

55. Cappell KM, Sherry RM, Yang JC, Goff SL, Vanasse DA, McIntyre L, et al. Long-Term Follow-Up of Anti-CD19 Chimeric Antigen Receptor T-Cell Therapy. J Clin Oncol. 2020;38(32):3805-15.

56. Shah NN, Johnson BD, Schneider D, Zhu F, Szabo A, Keever-Taylor CA, et al. Bispecific anti-CD20, anti-CD19 CAR T cells for relapsed B cell malignancies: a phase 1 dose escalation and expansion trial. Nat Med. 2020;26(10):1569-75.

57. Brudno JN, Lam N, Vanasse D, Shen YW, Rose JJ, Rossi J, et al. Safety and feasibility of anti-CD19 CAR T cells with fully human binding domains in patients with B-cell lymphoma. Nat Med. 2020;26(2):270-80.

58. Wang M, Munoz J, Goy A, Locke FL, Jacobson CA, Hill BT, et al. KTE-X19 CAR T-Cell Therapy in Relapsed or Refractory Mantle-Cell Lymphoma. N Engl J Med. 2020;382(14):1331-42.

59. Zhang X, Lu XA, Yang J, Zhang G, Li J, Song L, et al. Efficacy and safety of anti-CD19 CAR T-cell therapy in 110 patients with B-cell acute lymphoblastic leukemia with high-risk features. Blood Adv. 2020;4(10):2325-38.

60. Osborne W, Marzolini M, Tholouli E, Ramakrishnan A, Bachier CR, McSweeney PA, et al. Phase I Alexander study of AUTO3, the first CD19/22 dual targeting CAR T cell therapy, with pembrolizumab in patients with relapsed/refractory (r/r) DLBCL. Journal of Clinical Oncology. 2020;38(15_suppl):8001-.

61. Dai H, Wu Z, Jia H, Tong C, Guo Y, Ti D, et al. Bispecific CAR-T cells targeting both CD19 and CD22 for therapy of adults with relapsed or refractory B cell acute lymphoblastic leukemia. J Hematol Oncol. 2020;13(1):30.

62. Frey NV, Shaw PA, Hexner EO, Pequignot E, Gill S, Luger SM, et al. Optimizing Chimeric Antigen Receptor T-Cell Therapy for Adults With Acute Lymphoblastic Leukemia. J Clin Oncol. 2020;38(5):415-22.

63. Zhou X, Tu S, Wang C, Huang R, Deng L, Song C, et al. Phase I Trial of Fourth-Generation Anti-CD19 Chimeric Antigen Receptor T Cells Against Relapsed or Refractory B Cell Non-Hodgkin Lymphomas. Front Immunol. 2020;11:564099.

64. Gauthier J, Bezerra ED, Hirayama AV, Fiorenza S, Sheih A, Chou CK, et al. Factors associated with outcomes after a second CD19-targeted CAR T-cell infusion for refractory B cell malignancies. Blood. 2020.

65. Ying Z, Yang H, Guo Y, Li W, Zou D, Zhou D, et al. Relmacabtagene autoleucel (relma-cel) CD19 CAR-T therapy for adults with heavily pretreated relapsed/refractory large B-cell lymphoma in China. Cancer Med. 2020.

66. Pan J, Zuo S, Deng B, Xu X, Li C, Zheng Q, et al. Sequential CD19-22 CAR T therapy induces sustained remission in children with r/r B-ALL. Blood. 2020;135(5):387-91.

67. An F, Wang H, Liu Z, Wu F, Zhang J, Tao Q, et al. Influence of patient characteristics on chimeric antigen receptor T cell therapy in B-cell acute lymphoblastic leukemia. Nat Commun. 2020;11(1):5928.

68. Itzhaki O, Jacoby E, Nissani A, Levi M, Nagler A, Kubi A, et al. Head-to-head comparison of in-house produced CD19 CAR-T cell in ALL and NHL patients. J Immunother Cancer. 2020;8(1).

69. Sesques P, Ferrant E, Safar V, Wallet F, Tordo J, Dhomps A, et al. Commercial anti-CD19 CAR T cell therapy for patients with relapsed/refractory aggressive B cell lymphoma in a European center. Am J Hematol. 2020;95(11):1324-33.

70. Tong C, Zhang Y, Liu Y, Ji X, Zhang W, Guo Y, et al. Optimized tandem CD19/CD20 CAR-engineered T cells in refractory/relapsed B-cell lymphoma. Blood. 2020;136(14):1632-44.

71. Wang N, Hu X, Cao W, Li C, Xiao Y, Cao Y, et al. Efficacy and safety of CAR19/22 T-cell cocktail therapy in patients with refractory/relapsed B-cell malignancies. Blood. 2020;135(1):17-27.

72. Nastoupil LJ, Jain MD, Feng L, Spiegel JY, Ghobadi A, Lin Y, et al. Standard-of-Care Axicabtagene Ciloleucel for Relapsed or Refractory Large B-Cell Lymphoma: Results From the US Lymphoma CAR T Consortium. Journal of Clinical Oncology. 2020;38(27):3119-28.

73. Sang W, Shi M, Yang J, Cao J, Xu L, Yan D, et al. Phase II trial of co-administration of CD19- and CD20-targeted chimeric antigen receptor T cells for relapsed and refractory diffuse large B cell lymphoma. Cancer Med. 2020;9(16):5827-38.

74. Abramson JS, Palomba ML, Gordon LI, Lunning MA, Wang M, Arnason J, et al. Lisocabtagene maraleucel for patients with relapsed or refractory large B-cell lymphomas (TRANSCEND NHL 001): a multicentre seamless design study. Lancet. 2020;396(10254):839-52.

75. Jacobson C, Chavez JC, Sehgal AR, William BM, Munoz J, Salles G, et al. Primary Analysis of Zuma-5: A Phase 2 Study of Axicabtagene Ciloleucel (Axi-Cel) in Patients with Relapsed/Refractory (R/R) Indolent Non-Hodgkin Lymphoma (iNHL). Blood. 2020:<https://ashpublications.org/blood/article/136/Supplement%201/40/470462/Primary-Analysis-of-Zuma-5-A-Phase-2-Study-of> , accessed 15/1/2021.

76. Chen X, Li X, Liu Y, Zhang Z, Zhang X, Huang J, et al. A Phase I clinical trial of chimeric antigen receptor-modified T cells in patients with relapsed and refractory lymphoma. Immunotherapy. 2020;12(10):681-96.

77. Jacobson CA, Locke FL, Miklos DB, Herrera AF, Westin JR, Lee J, et al. End of Phase 1 Results from Zuma-6: Axicabtagene Ciloleucel (Axi-Cel) in Combination with Atezolizumab for the Treatment of Patients with Refractory Diffuse Large B Cell Lymphoma. Blood. 2018;132(Supplement 1):4192-.

78. Jacobson CA, Westin JR, Miklos DB, Herrera AF, Lee J, Seng J, et al. Phase 1/2 primary analysis of ZUMA-6: Axicabtagene ciloleucel (Axi-Cel) in combination With atezolizumab (Atezo) for the treatment of patients (Pts) with refractory diffuse large B cell lymphoma (DLBCL). Cancer Res. 2020;80:Abstract nr CT055.

79. <https://www.globenewswire.com/news-release/2020/12/05/2140158/0/en/Autolus-Therapeutics-presents-compelling-AUTO1-data-from-ALLCAR-Phase-1-study-in-Adult-Acute-Lymphoblastic-Leukemia-ALL-during-the-62nd-ASH-Annual-Meeting.html>, accessed 8.1.21.

80. Neelapu SS, Dickinson M, Ulrickson ML, Oluwole OO, Herrera AF, Thieblemont C, et al. Interim Analysis of ZUMA-12: A Phase 2 Study of Axicabtagene Ciloleucel (Axi-Cel) as First-Line Therapy in Patients (Pts) With High-Risk Large B Cell Lymphoma (LBCL). Blood. 2020:<https://ash.confex.com/ash/2020/webprogram/Paper134449.html> , accessed 16/01/2021

.

81. Ali SA, Shi V, Maric I, Wang M, Stroncek DF, Rose JJ, et al. T cells expressing an anti-B-cell maturation antigen chimeric antigen receptor cause remissions of multiple myeloma. Blood. 2016;128(13):1688-700.

82. Wang CM, Wu ZQ, Wang Y, Guo YL, Dai HR, Wang XH, et al. Autologous T Cells Expressing CD30 Chimeric Antigen Receptors for Relapsed or Refractory Hodgkin Lymphoma: An Open-Label Phase I Trial. Clin Cancer Res. 2017;23(5):1156-66.

83. Ramos CA, Ballard B, Zhang H, Dakhova O, Gee AP, Mei Z, et al. Clinical and immunological responses after CD30-specific chimeric antigen receptor-redirected lymphocytes. J Clin Invest. 2017;127(9):3462-71.

84. Mailankody S, Htut M, Lee K, Bensinger W, Devries T, Piasecki J, et al. JCARH125, Anti-BCMA CAR T-cell Therapy for Relapsed/Refractory Multiple Myeloma: Initial Proof of Concept Results from a Phase 1/2 Multicenter Study (EVOLVE). Blood. 2018;132:957-.

85. Mailankody S, Ghosh A, Staehr M, Purdon TJ, Roshal M, Halton E, et al. Clinical Responses and Pharmacokinetics of MCARH171, a Human-Derived Bcma Targeted CAR T Cell Therapy in Relapsed/Refractory Multiple Myeloma: Final Results of a Phase I Clinical Trial. Blood. 2018;132(Supplement 1):959-.

86. Garfall AL, Stadtmauer EA, Hwang W-T, Lacey SF, Melenhorst JJ, Krevvata M, et al. Anti-CD19 CAR T cells with high-dose melphalan and autologous stem cell transplantation for refractory multiple myeloma. JCI Insight. 2019;3(8).

87. Sallman DA, Kerre T, Poire X, Havelange V, Lewalle P, Davila ML, et al. Remissions in Relapse/Refractory Acute Myeloid Leukemia Patients Following Treatment with NKG2D CAR-T Therapy without a Prior Preconditioning Chemotherapy. Blood. 2018;132(Supplement 1):902-.

88. Liu Y, Chen Z, Fang H, Wei R, Yu K, Jiang S, et al. Durable Remission Achieved from Bcma-Directed CAR-T Therapy Against Relapsed or Refractory Multiple Myeloma. Blood. 2018;132(Supplement 1):956-.

89. Green DJ, Pont M, Sather BD, Cowan AJ, Turtle CJ, Till BG, et al. Fully Human Bcma Targeted Chimeric Antigen Receptor T Cells Administered in a Defined Composition Demonstrate Potency at Low Doses in Advanced Stage High Risk Multiple Myeloma. Blood. 2018;132(Supplement 1):1011-.

90. Li C, Wang Q, Zhu H, Mao X, Wang Y, Zhang Y, et al. T Cells Expressing Anti B-Cell Maturation Antigen Chimeric Antigen Receptors for Plasma Cell Malignancies. Blood. 2018;132:1013-.

91. Shi X, Yan L, Shang J, Qu S, Kang L, Zhou J, et al. Tandom Autologous Transplantation and Combined Infusion of CD19 and Bcma-Specific Chimeric Antigen Receptor T Cells for High Risk MM: Initial Safety and Efficacy Report from a Clinical Pilot Study. Blood. 2018;132(Supplement 1):1009-.

92. Raje N, Berdeja J, Lin Y, Siegel D, Jagannath S, Madduri D, et al. Anti-BCMA CAR T-Cell Therapy bb2121 in Relapsed or Refractory Multiple Myeloma. N Engl J Med. 2019;380(18):1726-37.

93. Yan Z, Cao J, Cheng H, Qiao J, Zhang H, Wang Y, et al. A combination of humanised anti-CD19 and anti-BCMA CAR T cells in patients with relapsed or refractory multiple myeloma: a single-arm, phase 2 trial. Lancet Haematol. 2019;6(10):e521-e9.

94. Baumeister SH, Murad J, Werner L, Daley H, Trebeden-Negre H, Gicobi JK, et al. Phase I Trial of Autologous CAR T Cells Targeting NKG2D Ligands in Patients with AML/MDS and Multiple Myeloma. Cancer Immunol Res. 2019;7(1):100-12.

95. <https://celyad.com/wp-content/uploads/2020/06/191209-Interim-Results-DEPLETHINK-%C2%B0POSTER.pdf>. (accessed Dec 21st 2020)

96. Li C, Mei H, Hu Y, Guo T, Liu L, Jiang H, et al. A Bispecific CAR-T Cell Therapy Targeting Bcma and CD38 for Relapsed/Refractory Multiple Myeloma: Updated Results from a Phase 1 Dose-Climbing Trial. Blood. 2019:<https://ashpublications.org/blood/article/134/Supplement_1/930/427103/A-Bispecific-CAR-T-Cell-Therapy-Targeting-Bcma-and> , accessed 16/1/2021.

97. Cohen AD, Garfall AL, Stadtmauer EA, Melenhorst JJ, Lacey SF, Lancaster E, et al. B cell maturation antigen-specific CAR T cells are clinically active in multiple myeloma. J Clin Invest. 2019;129(6):2210-21.

98. Li C, Zhou J, Wang J, Hu G, Du A, Zhou X, et al. Clinical responses and pharmacokinetics of fully human BCMA targeting CAR T-cell therapy in relapsed/refractory multiple myeloma. Journal of Clinical Oncology. 2019:<https://ascopubs.org/doi/abs/10.1200/JCO.2019.37.15_suppl.8013> , accessed 16/01/2021.

99. Chen L, Xu J, Fu W, Jin S, Yang S, Yan S, et al. Updated Phase 1 Results of a First-in-Human Open-Label Study of Lcar-B38M, a Structurally Differentiated Chimeric Antigen Receptor T (CAR-T) Cell Therapy Targeting B-Cell Maturation Antigen (Bcma). Blood. 2019;134:1858-.

100. Xu J, Chen LJ, Yang SS, Sun Y, Wu W, Liu YF, et al. Exploratory trial of a biepitopic CAR T-targeting B cell maturation antigen in relapsed/refractory multiple myeloma. Proc Natl Acad Sci U S A. 2019;116(19):9543-51.

101. Zhao WH, Liu J, Wang BY, Chen YX, Cao XM, Yang Y, et al. A phase 1, open-label study of LCAR-B38M, a chimeric antigen receptor T cell therapy directed against B cell maturation antigen, in patients with relapsed or refractory multiple myeloma. J Hematol Oncol. 2018;11(1):141.

102. Yan L, Yan Z, Shang J, Shi X, Jin S, Kang L, et al. Sequential CD19- and Bcma-Specific Chimeric Antigen Receptor T Cell Treatment for RRMM: Report from a Single Center Study. Blood. 2019;134(Supplement_1):578-.

103. Cowan AJ, Pont M, Sather BD, Turtle CJ, Till BG, Nagengast AM, et al. Efficacy and Safety of Fully Human Bcma CAR T Cells in Combination with a Gamma Secretase Inhibitor to Increase Bcma Surface Expression in Patients with Relapsed or Refractory Multiple Myeloma. Blood. 2019;134(Supplement_1):204-.

104. Jie J, Hao S, Jiang S, Li Z, Yang M, Zhang W, et al. Phase 1 Trial of the Safety and Efficacy of Fully Human Anti-Bcma CAR T Cells in Relapsed/Refractory Multiple Myeloma. Blood. 2019;134(Supplement_1):4435-.

105. Popat R, Zweegman S, Cavet J, Yong K, Lee L, Faulkner J, et al. Phase 1 First-in-Human Study of AUTO2, the First Chimeric Antigen Receptor (CAR) T Cell Targeting APRIL for Patients with Relapsed/Refractory Multiple Myeloma (RRMM). Blood. 2019;134(Supplement_1):3112-.

106. Mikkilineni L, Manasanch EE, Lam N, Vanasse D, Brudno JN, Maric I, et al. T Cells Expressing an Anti-B-Cell Maturation Antigen (BCMA) Chimeric Antigen Receptor with a Fully-Human Heavy-Chain-Only Antigen Recognition Domain Induce Remissions in Patients with Relapsed Multiple Myeloma. Blood. 2019;134(Supplement_1):3230-.

107. Berdeja JG, Alsina M, Shah ND, Siegel DS, Jagannath S, Madduri D, et al. Updated Results from an Ongoing Phase 1 Clinical Study of bb21217 Anti-Bcma CAR T Cell Therapy. Blood. 2019;134(Supplement_1):927-.

108. Costello CL, Cohen AD, Patel KK, Ali SS, Berdeja JG, Shah N, et al. Phase 1/2 Study of the Safety and Response of P-BCMA-101 CAR-T Cells in Patients with Relapsed/Refractory (r/r) Multiple Myeloma (MM) (PRIME) with Novel Therapeutic Strategies. Blood. 2020:<https://ash.confex.com/ash/2020/webprogram/Paper142695.html>, accessed 9/1/21.

109. Deeren D, Maertens JA, Lin T, Beguin Y, Demoulin B, Fontaine M, et al. First Results from the Dose Escalation Segment of the Phase I Clinical Study Evaluating Cyad-02, an Optimized Non Gene-Edited Engineered NKG2D CAR T-Cell Product, in Relapsed or Refractory Acute Myeloid Leukemia and Myelodysplastic Syndrome Patients. Blood. 2020;136(Supplement 1):36-.

110. <https://www.globenewswire.com/news-release/2020/12/07/2140208/0/en/Celyad-Oncology-Provides-Updates-on-Allogeneic-and-Autologous-CAR-T-Programs-at-62nd-ASH-Annual-Meeting-and-Exposition.html>, analysed 11/1/2021.

111. Chen W, Fu C, Cai Z, Li Z, Wang H, Yan L, et al. Results from Lummicar-1: A Phase 1 Study of Fully Human B-Cell Maturation Antigen-Specific CAR T Cells (CT053) in Chinese Subjects with Relapsed and/or Refractory Multiple Myeloma. Blood. 2020:<https://ash.confex.com/ash/2020/webprogram/Paper140727.html>, accessed 9/1/2021.

112. Kumar SK, Baz RC, Orlowski RZ, Anderson LD, Ma H, Shrewsbury A, et al. Results from Lummicar-2: A Phase 1b/2 Study of Fully Human B-Cell Maturation Antigen-Specific CAR T Cells (CT053) in Patients with Relapsed and/or Refractory Multiple Myeloma. Blood. 2020:<https://ash.confex.com/ash/2020/webprogram/Paper139802.html>, accessed 9/1/2021.

113. Munshi NC, Anderson LD, Shah N, Jagannath S, Berdeja JG, Lonial S, et al. Idecabtagene vicleucel (ide-cel; bb2121), a BCMA-targeted CAR T-cell therapy, in patients with relapsed and refractory multiple myeloma (RRMM): Initial KarMMa results. Journal of Clinical Oncology. 2020:<https://ascopubs.org/doi/abs/10.1200/JCO.2020.38.15_suppl.8503> , accessed 16/01/2021.

114. Berdeja JG, Madduri D, Usmani SZ, Singh I, Zudaire E, Yeh T-M, et al. Update of CARTITUDE-1: A phase Ib/II study of JNJ-4528, a B-cell maturation antigen (BCMA)-directed CAR-T-cell therapy, in relapsed/refractory multiple myeloma. Journal of Clinical Oncology. 2020;38(15_suppl):8505-.

115. Madduri D, Berdeja JG, Usmani SZ, Jakubowiak A, Agha M, Cohen AD, et al. CARTITUDE-1: Phase 1b/2 Study of Ciltacabtagene Autoleucel, a B-Cell Maturation Antigen–Directed Chimeric Antigen Receptor T Cell Therapy, in Relapsed/Refractory Multiple Myeloma. Blood. 2020:<https://ash.confex.com/ash/2020/webprogram/Paper136307.html>, accessed 11/1/2021.

116. Warren RS FG, Bergsland EK, Pennathur-Das R, Nemunaitis J, Venook AP, Hege KM Clinical studies of regional and systemic gene therapy with autologous CC49-z modified T cells in colorectal cancer metastatic to the liver. . Cancer Gene Therapy. 1998;5:S1-S2.

117. Ma Q, Gonzalo-Daganzo RM, Junghans RP. Genetically engineered T cells as adoptive immunotherapy of cancer. Cancer Chemother Biol Response Modif. 2002;20:315-41.

118. Hege KM, Bergsland EK, Fisher GA, Nemunaitis JJ, Warren RS, McArthur JG, et al. Safety, tumor trafficking and immunogenicity of chimeric antigen receptor (CAR)-T cells specific for TAG-72 in colorectal cancer. J Immunother Cancer. 2017;5:22.

119. Kershaw MH, Westwood JA, Parker LL, Wang G, Eshhar Z, Mavroukakis SA, et al. A phase I study on adoptive immunotherapy using gene-modified T cells for ovarian cancer. Clin Cancer Res. 2006;12(20 Pt 1):6106-15.

120. Park JR, Digiusto DL, Slovak M, Wright C, Naranjo A, Wagner J, et al. Adoptive transfer of chimeric antigen receptor re-directed cytolytic T lymphocyte clones in patients with neuroblastoma. Mol Ther. 2007;15(4):825-33.

121. Lamers CH, Sleijfer S, Vulto AG, Kruit WH, Kliffen M, Debets R, et al. Treatment of metastatic renal cell carcinoma with autologous T-lymphocytes genetically retargeted against carbonic anhydrase IX: first clinical experience. J Clin Oncol. 2006;24(13):e20-2.

122. Lamers CH, Willemsen R, van Elzakker P, van Steenbergen-Langeveld S, Broertjes M, Oosterwijk-Wakka J, et al. Immune responses to transgene and retroviral vector in patients treated with ex vivo-engineered T cells. Blood. 2011;117(1):72-82.

123. Lamers CH, Sleijfer S, van Steenbergen S, van Elzakker P, van Krimpen B, Groot C, et al. Treatment of metastatic renal cell carcinoma with CAIX CAR-engineered T cells: clinical evaluation and management of on-target toxicity. Mol Ther. 2013;21(4):904-12.

124. Pule MA, Savoldo B, Myers GD, Rossig C, Russell HV, Dotti G, et al. Virus-specific T cells engineered to coexpress tumor-specific receptors: persistence and antitumor activity in individuals with neuroblastoma. Nat Med. 2008;14(11):1264-70.

125. Louis CU, Savoldo B, Dotti G, Pule M, Yvon E, Myers GD, et al. Antitumor activity and long-term fate of chimeric antigen receptor-positive T cells in patients with neuroblastoma. Blood. 2011;118(23):6050-6.

126. Slovin SF, Wang X, Hullings M, Arauz G, Bartido S, Lewis JS, et al. Chimeric antigen receptor (CAR+) modified T cells targeting prostate-specific membrane antigen (PSMA) in patients (pts) with castrate metastatic prostate cancer (CMPC). Journal of Clinical Oncology. 2013:<https://ascopubs.org/doi/10.1200/jco.2013.31.6_suppl.72> , accessed 16/01/21.

127. Ahmed N, Brawley VS, Hegde M, Robertson C, Ghazi A, Gerken C, et al. Human Epidermal Growth Factor Receptor 2 (HER2) -Specific Chimeric Antigen Receptor-Modified T Cells for the Immunotherapy of HER2-Positive Sarcoma. J Clin Oncol. 2015;33(15):1688-96.

128. Katz SC, Burga RA, McCormack E, Wang LJ, Mooring W, Point GR, et al. Phase I Hepatic Immunotherapy for Metastases Study of Intra-Arterial Chimeric Antigen Receptor-Modified T-cell Therapy for CEA+ Liver Metastases. Clin Cancer Res. 2015;21(14):3149-59.

129. Feng K, Guo Y, Dai H, Wang Y, Li X, Jia H, et al. Chimeric antigen receptor-modified T cells for the immunotherapy of patients with EGFR-expressing advanced relapsed/refractory non-small cell lung cancer. Sci China Life Sci. 2016;59(5):468-79.

130. Guo Y, Feng K, Liu Y, Wu Z, Dai H, Yang Q, et al. Phase I Study of Chimeric Antigen Receptor-Modified T Cells in Patients with EGFR-Positive Advanced Biliary Tract Cancers. Clin Cancer Res. 2018;24(6):1277-86.

131. Thistlethwaite FC, Gilham DE, Guest RD, Rothwell DG, Pillai M, Burt DJ, et al. The clinical efficacy of first-generation carcinoembryonic antigen (CEACAM5)-specific CAR T cells is limited by poor persistence and transient pre-conditioning-dependent respiratory toxicity. Cancer Immunol Immunother. 2017;66(11):1425-36.

132. Zhang C, Wang Z, Yang Z, Wang M, Li S, Li Y, et al. Phase I Escalating-Dose Trial of CAR-T Therapy Targeting CEA(+) Metastatic Colorectal Cancers. Mol Ther. 2017;25(5):1248-58.

133. Tchou J, Zhao Y, Levine BL, Zhang PJ, Davis MM, Melenhorst JJ, et al. Safety and Efficacy of Intratumoral Injections of Chimeric Antigen Receptor (CAR) T Cells in Metastatic Breast Cancer. Cancer Immunol Res. 2017;5(12):1152-61.

134. Heczey A, Louis CU, Savoldo B, Dakhova O, Durett A, Grilley B, et al. CAR T Cells Administered in Combination with Lymphodepletion and PD-1 Inhibition to Patients with Neuroblastoma. Mol Ther. 2017;25(9):2214-24.

135. O'Rourke DM, Nasrallah MP, Desai A, Melenhorst JJ, Mansfield K, Morrissette JJD, et al. A single dose of peripherally infused EGFRvIII-directed CAR T cells mediates antigen loss and induces adaptive resistance in patients with recurrent glioblastoma. Sci Transl Med. 2017;9(399).

136. Ahmed N, Brawley V, Hegde M, Bielamowicz K, Kalra M, Landi D, et al. HER2-Specific Chimeric Antigen Receptor-Modified Virus-Specific T Cells for Progressive Glioblastoma: A Phase 1 Dose-Escalation Trial. JAMA Oncol. 2017;3(8):1094-101.

137. Feng K, Liu Y, Guo Y, Qiu J, Wu Z, Dai H, et al. Phase I study of chimeric antigen receptor modified T cells in treating HER2-positive advanced biliary tract cancers and pancreatic cancers. Protein Cell. 2018;9(10):838-47.

138. Beatty GL, O'Hara MH, Lacey SF, Torigian DA, Nazimuddin F, Chen F, et al. Activity of Mesothelin-Specific Chimeric Antigen Receptor T Cells Against Pancreatic Carcinoma Metastases in a Phase 1 Trial. Gastroenterology. 2018;155(1):29-32.

139. Papa S, Adami A, Metoudi M, Achkova D, van Schalkwyk M, Parente-Pereira A, et al. A phase I trial of T4 CAR T-cell immunotherapy in head and neck squamous cancer (HNSCC). J Clin Oncol. 2018;<https://ascopubs.org/doi/abs/10.1200/JCO.2018.36.15_suppl.3046>.

140. Haas AR, Tanyi JL, O'Hara MH, Gladney WL, Lacey SF, Torigian DA, et al. Phase I Study of Lentiviral-Transduced Chimeric Antigen Receptor-Modified T Cells Recognizing Mesothelin in Advanced Solid Cancers. Mol Ther. 2019;27(11):1919-29.

141. Becerra CR, Hoof P, Paulson AS, Manji GA, Gardner O, Malankar A, et al. Ligand-inducible, prostate stem cell antigen (PSCA)-directed GoCAR-T cells in advanced solid tumors: Preliminary results from a dose escalation. Journal of Clinical Oncology. 2019;37(4_suppl):283-.

142. Navai S, Derenzo C, Joseph S, Sanber K, Byrd T, Zhang H, et al. Administration of HER2-CAR T cells after lymphodepletion safely improves T cell expansion and induces clinical responses in patients with advanced sarcomas. 2019:<https://www.abstractsonline.com/pp8/#!/6812/presentation/9413> accessed 13/1/2021.

143. Hegde M, Joseph SK, Pashankar F, DeRenzo C, Sanber K, Navai S, et al. Tumor response and endogenous immune reactivity after administration of HER2 CAR T cells in a child with metastatic rhabdomyosarcoma. Nat Commun. 2020;11(1):3549.

144. Narayan V, Gladney W, Plesa G, Vapiwala N, Carpenter E, Maude SL, et al. A phase I clinical trial of PSMA-directed/TGFβ-insensitive CAR-T cells in metastatic castration-resistant prostate cancer. Journal of Clinical Oncology. 2019;37(7_suppl):TPS347-TPS.

145. <https://www.morressier.com/article/phase-1-clinical-trial-psmadirectedtgfinsensitive-cart-cells-metastatic-castrationresistant-prostate-cancer/5f69edb69b74b699bf38c603>? a.

146. Zhan X, Wang B, Li Z, Li J, Wang H, Chen L, et al. Phase I trial of Claudin 18.2-specific chimeric antigen receptor T cells for advanced gastric and pancreatic adenocarcinoma. J Clin Oncol. 2019:<https://ascopubs.org/doi/abs/10.1200/JCO.2019.37.15_suppl.509> accessed 13/1/21.

147. Adusumilli PS, Zauderer MG, Rusch VW, O’Cearbhaill R, Zhu A, Ngai D, et al. Regional delivery of mesothelin-targeted CAR T cells for pleural cancers: Safety and preliminary efficacy in combination with anti-PD-1 agent. Journal of Clinical Oncology. 2019:<https://ascopubs.org/doi/abs/10.1200/JCO.2019.37.15_suppl.511> accessed 13/1/21.

148. Goff SL, Morgan RA, Yang JC, Sherry RM, Robbins PF, Restifo NP, et al. Pilot Trial of Adoptive Transfer of Chimeric Antigen Receptor-transduced T Cells Targeting EGFRvIII in Patients With Glioblastoma. J Immunother. 2019;42(4):126-35.

149. Guo JX, Wu CX, Wang PF, Li ZJ, Han S, Jin W, et al. Bioactivity and safety of chimeric switch receptor T cells in glioblastoma patients. Front Biosci (Landmark Ed). 2019;24:1158-66.

150. Katz SC, Hardaway J, Prince E, Guha P, Cunetta M, Moody A, et al. HITM-SIR: phase Ib trial of intraarterial chimeric antigen receptor T-cell therapy and selective internal radiation therapy for CEA(+) liver metastases. Cancer Gene Ther. 2020;27(5):341-55.

151. Shi D, Shi Y, Kaseb AO, Qi X, Zhang Y, Chi J, et al. Chimeric Antigen Receptor-Glypican-3 T-Cell Therapy for Advanced Hepatocellular Carcinoma: Results of Phase I Trials. Clin Cancer Res. 2020;26(15):3979-89.

152. Straathof K, Flutter B, Wallace R, Jain N, Loka T, Depani S, et al. Antitumor activity without on-target off-tumor toxicity of GD2-chimeric antigen receptor T cells in patients with neuroblastoma. Sci Transl Med. 2020;12(571).

153. O’Cearbhaill RE, Park JH, Halton EF, Diamonte CR, Mead E, Lakhman Y, et al. Gynecologic Oncology. 2020:<https://www.gynecologiconcology-online.net/article/S0090-8258(20)31854-0/fulltext>, accessed 13/1/2021.

154. Cruz CR, Micklethwaite KP, Savoldo B, Ramos CA, Lam S, Ku S, et al. Infusion of donor-derived CD19-redirected virus-specific T cells for B-cell malignancies relapsed after allogeneic stem cell transplant: a phase 1 study. Blood. 2013;122(17):2965-73.

155. Kochenderfer JN, Dudley ME, Carpenter RO, Kassim SH, Rose JJ, Telford WG, et al. Donor-derived CD19-targeted T cells cause regression of malignancy persisting after allogeneic hematopoietic stem cell transplantation. Blood. 2013;122(25):4129-39.

156. Brudno JN, Somerville RP, Shi V, Rose JJ, Halverson DC, Fowler DH, et al. Allogeneic T Cells That Express an Anti-CD19 Chimeric Antigen Receptor Induce Remissions of B-Cell Malignancies That Progress After Allogeneic Hematopoietic Stem-Cell Transplantation Without Causing Graft-Versus-Host Disease. J Clin Oncol. 2016;34(10):1112-21.

157. Rossig C, Pule M, Altvater B, Saiagh S, Wright G, Ghorashian S, et al. Vaccination to improve the persistence of CD19CAR gene-modified T cells in relapsed pediatric acute lymphoblastic leukemia. Leukemia. 2017;31(5):1087-95.

158. <https://celyad.com/wp-content/uploads/2020/06/191109-SITC2019_poster_P330_ALLOSHRINK-%C2%B0POSTER.pdf>. (accessed Dec 21st 2020)

159. <https://www.businesswire.com/news/home/20210117005022/en/Celyad-Oncology-Presents-Data-Update-from-Phase-1-alloSHRINK-Trial-for-CYAD-101-in-mCRC-at-ASCO-GI-Symposium> a.

160. <https://www.globenewswire.com/news-release/2020/05/29/2040891/0/en/Allogene-Therapeutics-with-Collaborator-Servier-Reports-Positive-Results-from-its-Phase-1-ALPHA-Study-of-ALLO-501-in-Relapsed-Refractory-Non-Hodgkin-Lymphoma-at-the-American-Societ.html>, accessed 11/1/2021.

161. Neelapu SS, Munoz J, Locke FL, Miklos DB, Brown R, McDevitt JT, et al. First-in-human data of ALLO-501 and ALLO-647 in relapsed/refractory large B-cell or follicular lymphoma (R/R LBCL/FL): ALPHA study. J Clin Oncol. 2020:<https://ascopubs.org/doi/abs/10.1200/JCO.2020.38.15_suppl.8002>, accessed 11/1/2021.

162. <https://ir.allogene.com/news-releases/news-release-details/allogene-therapeutics-reports-positive-initial-results-phase-1>. (accessed Dec 21st 2020)

163. Mailankody S, Matous JV, Liedtke M, Sidana S, Malik S, Nath R, et al. Universal: An Allogeneic First-in-Human Study of the Anti-Bcma ALLO-715 and the Anti-CD52 ALLO-647 in Relapsed/Refractory Multiple Myeloma. Blood. 2020:<https://ash.confex.com/ash/2020/webprogram/Paper140641.html>, accessed 11/1/2021.

164. <https://www.globenewswire.com/news-release/2020/10/21/2111729/0/en/CRISPR-Therapeutics-Reports-Positive-Top-Line-Results-from-Its-Phase-1-CARBON-Trial-of-CTX110-in-Relapsed-or-Refractory-CD19-B-cell-Malignancies.html>. (accessed Dec 21st 2020)

165. Liu E, Marin D, Banerjee P, Macapinlac HA, Thompson P, Basar R, et al. Use of CAR-Transduced Natural Killer Cells in CD19-Positive Lymphoid Tumors. N Engl J Med. 2020;382(6):545-53.

166. Benjamin R, Graham C, Yallop D, Jozwik A, Mirci-Danicar OC, Lucchini G, et al. Genome-edited, donor-derived allogeneic anti-CD19 chimeric antigen receptor T cells in paediatric and adult B-cell acute lymphoblastic leukaemia: results of two phase 1 studies. Lancet. 2020;396(10266):1885-94.
